# ﻿Integrative analysis reveals cryptic speciation linked to habitat differentiation within Albanian populations of the anomalous blues (Lepidoptera, Lycaenidae, *Polyommatus* Latreille, 1804)

**DOI:** 10.3897/CompCytogen.v16.i4.90558

**Published:** 2022-11-15

**Authors:** Laurian Parmentier, Roger Vila, Vladimir Lukhtanov

**Affiliations:** 1 Department of Plants & Crops, Lab Agrozoology, Ghent University, Coupure Links 653, 9000, Ghent, Belgium Ghent University Ghent Belgium; 2 Flemish Entomological Society, Workgroup Butterflies, Moerbeekstraat 29, 9870, Zulte, Belgium Flemish Entomological Society, Workgroup Butterflies Zulte Belgium; 3 Institut de Biologia Evolutiva (CSIC-Universitat Pompeu Fabra), Passeig Marítim de la Barceloneta 37, 08003, Barcelona, Spain CSIC-Universitat Pompeu Fabra Barcelona Spain; 4 Department of Karyosystematics, Zoological Institute of Russian Academy of Sciences, Universitetskaya nab. 1, 199034 Saint Petersburg, Russia Zoological Institute of Russian Academy of Sciences Saint Petersburg Russia

**Keywords:** biodiversity, chromosome number, *COI*, conservation, DNA barcoding, karyotype, mitochondrial marker, protected species, wing colour morphometrics

## Abstract

The Balkan Peninsula is one of the greatest hotspots for biodiversity in Europe. While the region has been investigated thoroughly, some parts remain understudied and may still harbour undiscovered diversity, even in well-studied organisms such as Lepidoptera. Here we investigated the group of the so-called anomalous blue butterflies, also known as ‘brown complex’ of the subgenus Agrodiaetus Hübner, 1822 including the taxa of the entire *Polyommatusaroaniensis* (Brown, 1976) species complex. This species complex is distributed in the southern part of the Balkan Peninsula and known to be represented by three closely related allopatric species, differentiated by their chromosome numbers (n) and mitochondrial (mt) DNA. These are *P.aroaniensis* sensu stricto (Southern Greece, Peloponnese, n=47–48; mt haplogroup *aroa1*), *P.timfristos* Lukhtanov, Vishnevskaya et Shapoval, 2016 (Central Greece, Attika, n=38, *aroa2*) and *P.orphicus* Kolev, 2005 (North-Eastern Greece, Southern Bulgaria, n=41–42, *orph1*).

Based on an analysis of chromosomal, molecular and morphological markers, we demonstrate that a fourth taxon of this species complex exists in Albania. This taxon possesses the mt haplogroup *aroa3*, which is the most differentiated within the entire *P.aroaniensis* species complex, and the karyotype (n=42–43), which differs by one fixed chromosome fission from *P.orphicus*. The Albanian taxon seems to be ecologically specialised (habitat on dark-coloured, ophiolitic substrate soils) and differs in colouration (wing reflectance) from the others taxa of the *P.aroaniensis* species group. Based on the evidence here presented and following the current view of the taxonomy of the group, we propose considering the Albanian taxon as a new species, here described as *Polyommatuslurae***sp. nov.** At the contact zone between the new species and *P.orphicus*, in addition to typical ones, we detected specimens with haplogroup orph2, karyotype n=43 and intermediate morphology, which seem to represent *P.lurae* × *P.orphicus* hybrids.

## ﻿Introduction

The so-called anomalous blues of the subgenus Agrodiaetus Hübner, 1822 constitute a distinct lineage within the species-rich genus *Polyommatus* Latreille, 1804 (Talavera et al. 2013a). The subgenus Agrodiaetus is distributed throughout the Western Palaearctic and Central Asia. With over 120 species described, this subgenus is a striking example of explosive radiation in the last three million years (Kandul et al. 2004; Kandul et al. 2007; Talavera et al. 2013b). Sexes are often dimorphic, with females usually brown in colour, and with males displaying colours such as blue, white, silver, orange, violet, or brown on the dorsal part of the wings, which is probably a signature of the reinforcement of pre-zygotic isolation (Lukhtanov et al. 2005; Dincă et al. 2017).

In Europe, most *Agrodiaetus* species are restricted to the southern warm parts of the continent, and most of them are endemic to relatively small areas. The Balkans are one of the richest European regions for subgenus Agrodiaetus (Kudrna et al. 2015) and, currently, the following taxa of *Agrodiaetus* with brown males are recognised as present in this region: *Polyommatusadmetus* (Esper, 1783), *Polyommatusripartii* (Freyer, 1830) and those under the so-called *Polyommatusaroaniensis* Brown, 1976 species complex. In this paper, we focus on the latter species complex, which remains still insufficiently studied, especially in underexplored regions like Albania.

The first species of “brown” *Agrodiaetus* described as endemic to the Balkans was *P.aroaniensis* (Brown, 1976). After Coutsis (1972) attracted attention to a population of “*P.ripartii*” on Mt. Chelmos in Southern Greece, noting that in “about half of the specimens the white streak on the hindwing underside, always present in *ripartii*, was completely absent”. Brown (1976) wrote about this population from Chelmos as an “unrecognized *Agrodiaetus* sp. similar and often sympatric with *ripartii* [*pelopi*] in Greece” (Kolev and van der Poorten 1997). The haploid chromosome number initially identified by Brown (1976) as n=15–16 was later corrected to n=47–48 (Lukhtanov et al. 2003). Apart from the unique chromosome number, typical *P.aroaniensis* is characterized by a coffee-brown colour with a perceptible reddish hue and by the lack of darker marks along the margins. While *P.aroaniensis* was apparently first discovered based on the absent or vestigial white streaks on the hindwings, it was later discussed that this trait was only valuable for about 60% of the specimens in the population (Kolev and van der Poorten 1997). Using the white stripe state (absent, reduced or prominent presence) as an identification trait for *P.aroaniensis* turned out to be difficult, as has been recently shown by the misidentification of *P.aroaniensis* in Croatia. Indeed, some specimens lacking this white stripe were initially determined as *P.aroaniensis* based only on this morphological trait, and were later corrected into *P.ripartii* by the use of the mitochondrial gene *COI* for identification (Lovrenčić et al. 2016). Yet, the distribution of *P.aroaniensis* seems to be much broader and at present it is still not fully understood where the limits of distribution lie, especially for the northern distribution range in Albania, North Macedonia and Bulgaria.

Then, almost three decades later, Lukhtanov and Wiemers (2003) described a new species of the brown *Agrodiaetus* from the extreme east of Turkey (Van province): *Polyommatusdantchenkoi* (Lukhtanov et Wiemers, 2003). About 2000 km to the west, another taxon with the same chromosome number (n = 41–42) was found in the Bulgarian Rhodope Mountains in the border area with Greece. This new taxon was initially considered a subspecies of the Turkish taxon: *Polyommatusdantchenkoiorphicus* Kolev, 2005 (Kolev 2005), but is now recognized as a separate species following the latest nomenclature by Wiemers et al. (2018): *Polyommatusorphicus* Kolev, 2005. The relationship to the taxon *Polyommatuseleniae* Coutsis et J. de Prins, 2005 (Coutsis and De Prins 2005) described in the same year from Northern Greece in almost the same area is still somewhat confusing in the available literature. Tshikolovets (2011), who treats *P.orphicus* as a subspecies (*Polyommatusdantchenkoiorphicus*), writes about *P.eleniae*: “With the same chromosome number as *dantchenkoi* but differing in reduced marginal spots on the underside of hindwings.” In contrast, Kudrna et al. (2011) states: “*Polyommatusorphicus* is a newly discovered supposedly distinct species reported so far from Bulgaria (Sliven, Smolyan), Greece (Mt. Falakro) and Macedonia (Kozjak, Rudina, Asandjura) by Wiemers et al. (2009) and M. Wiemers (pers. comm.)”. And “*P.eleniae* Coutsis et Prins, 2005, is a junior subjective synonym of *P.orphicus*; the printed dates of publication being 01.12.2005 and 07.06.2005 respectively. Both taxa appear to be identical” (Kudrna et al. 2011; Kudrna 2019). While the markings of the withe stripe on the underside of the hindwing are less pronounced for *P.eleniae*, synonymy with *P.orphicus* has been proposed by Vishnevskaya et al. (2016) based on identical results of genetical markers, mitochondrial (*COI*) and nuclear (*ITS2*), and karyology (n=41–42). This view has been accepted in the checklist by Wiemers et al. (2018), which we follow in this paper.

The above shows that the systematics of the “brown” *Agrodiaetus* is complex. Indeed, more recently, Vishnevskaya et al. (2016) demonstrated that *P.aroaniensis* in Greece actually contained two cryptic species. By applying a combined analysis of mitochondrial and nuclear markers and karyotype, *P.timfristos* Lukhtanov, Vishnevskaya et Shapoval, 2016 was described from Mt. Timfristos and adjacent Parnassos mountains with a different karyotype (n=38) and mitochondrial haplogroup compared to *P.aroaniensis* s.s. In summary, the latest accepted systematics considers the presence of three species of the *Polyommatusaroaniensis* species complex in the Balkans: *P.aroaniensis* s.s., *P.orphicus* and *P.timfristos*.

Generally, identification of species in the subgenus Agrodiaetus remains challenging because of considerable geographic and individual variability in habitus (Dincă et al. 2013), e.g. the state of the white stripe on the underside of hindwings. However, modern identification techniques based on genetic markers in combination with karyological data, often obtain reliable differentiation between *Agrodiaetus* taxa (Lukhtanov and Dantchenko 2002; Wiemers 2003; Kandul et al. 2004; Lukhtanov et al. 2006; Vila et al. 2010). Besides, at species level, subtle fixed variations in traits such as wing colour are still useful for identification in the Agrodiaetus subgenus and other Polyommatus sp(p)., especially male dorsal wing colour (Lafranchis 2004). Indeed, new morphometric techniques based on measurements of wing colour and its reflectance pattern have been tested for species identification and delimitation. For example, remarkably good correlations with genetic markers have been found for *Lysandrabellargus* and *Polyommatusicarus* (Kertész et al. 2021; Piszter et al. 2021) and this colour morphometric technique can also be useful for discriminating between species of the *Agrodiaetus* subgenus.

Thus, an integrative analysis combining multiple markers and techniques may be the best solution to resolve complex systematics and to uncover potential cryptic diversity in the *Agrodiaetus* subgenus (Lukhtanov and Dantchenko 2017; Kertész et al. 2021). By using such an integrative approach, based on combined molecular, cytogenetic, and colour morphometric analyses, we here demonstrate that a fourth taxon within the entire *Polyommatusaroaniensis* species complex is present in Albania.

## ﻿Methods

### ﻿Sample collection and storage

All butterflies were collected by L. Parmentier at the different biotopes investigated in Albania, in the provinces of Korçë, Elbasan, Dibër and Kukës. Collected samples were put in glassine envelopes in the field, a unique code assigned to each and stored in cooled plastic boxes. At different sites, a selection of fresh male samples was kept alive, until the posterior part of the abdomen was removed for karyological analysis. Taking into account the possibility of multiple cryptic species within a local area even in well-studied European butterflies (Dincă et al. 2011; Hinojosa et al. 2022), multiple individuals were collected in each place, paying special attention to the specimens with unusual or intermediate morphology (Vishnevskaya et al. 2016). Unless otherwise noted, all collected samples were imagoes that were spread and dried to be used for the analysis of habitus, and all are stored in L. Parmentier’s private collection (LPAcoll, Zulte, Belgium). Legs used for DNA analysis are stored in R. Vila’s Collection (RVcoll, Institute of Evolutionary Biology, Barcelona, Spain) and karyotype plates in the Zoological Institute of the Russian Academy of Sciences. Pictures of biotopes were taken with an Iphone 6 and butterflies with a Canon 70D and a 100mm macro lens.

### ﻿DNA extraction and sequencing

In order to elucidate the genetics of the subgenus Agrodiaetus in Albania, we analysed DNA of 41 Albanian specimens (males and females). To put them in context, we mined 19 additional *COI* sequences from GenBank, a subset where the most similar sequences to the Albanian ones were included and overlapped at least 650 base pairs (bp) of the cytochrome c oxidase subunit (*COI*). DNA extraction was done following the protocol described in Dincă et al. (2017). The primers LepF1 and LepR1 (ATTCAACCAATCATAAAGATATTGG and TAAACTTCTGGATGTCCAAAAAATCA respectively) were employed for the *COI* amplification, obtaining a full DNA barcode fragment of 658 base pairs (bp). Double-stranded DNA was amplified in 25 μl volume reactions: 14.4 μl ultra-pure (HPLC quality) water, 5 μl 5X buffer, 2 μl 25mM MgCl_2_, 0.5 μl 10 mM dNTP, 0.5 μl of each primer (10 μM), 0.1 μl Taq DNA Polymerase (Promega) and 2 μl of extracted DNA. Conditions for the PCR cycles were set as follow: first denaturation step at 92 °C for 60 s, then 92 °C for 15 s, 48 °C for 45 s and 62 °C for 150 s in 5 cycles and other 30 cycles changing the annealing temperature to 52 °C with the final extension step at 62 °C for 7 min. A 411 to 440 bp fragment at the 5’ end of the nuclear *ITS2* was amplified by polymerase chain reaction using the primers ITS3 (GCATCGATGAAGAACGCAGC) and ITS4 (TCCTCCGCTTATTGATATGC) (White et al. 1990). The reactions were prepared as for *COI* but, in this case, the typical thermal cycling profile was: 95 °C for 45 s, 51 °C for 60 s and 72 °C for 60 s, for 40 cycles. PCR products were purified and Sanger sequenced by Macrogen Inc. Europe (Amsterdam, the Netherlands). All new *COI* and *ITS2* sequences have been deposited in GenBank (ON715895–ON715938 for *COI* and OP537924–OP537930 for *ITS2*) (Table 1).

**Table 1.** List of the studied samples of brown Polyommatus (Agrodiaetus) from Albania.

GenBank nr *COI* barcode	GenBank nr ITS2	LPA coll code	RV coll code	Karyo–type ID	Species	Sex	Chromo–some number	Mt haplo-group (lineage)	Locality	Remark biotope: soil type
ON715909	–	17-70-01	17E536	–	*P.orphicus*	F	–	*orph*2	Valikardhë region, Zerqan	Pure karst soil
ON715910	–	17-70-02	17F269	–	*P.orphicus*	M	–	*orph*2	Valikardhë region, Zerqan	Pure karst soil
ON715911	OP537928	18-70-K75	18D275	K75	*P.orphicus*	M	n=42	*orph*2	Valikardhë region, Zerqan	Pure karst soil
ON715912	–	18-111-K11	18D211	–	*P.orphicus*	M	–	*orph*2	Valikardhë	Pure karst soil
ON715913	–	18-111-K48	18D248	–	*P.orphicus*	M	–	*orph*2	Valikardhë	Pure karst soil
ON715914	–	18-111-K18	18D218	–	*P.orphicus*	M	–	*orph*2	Valikardhë	Pure karst soil
ON715915	–	18-111-K93	18D293	–	*P.orphicus*	M	–	*orph*2	Valikardhë	Pure karst soil
ON715916	–	18-111-K94	18D294	–	*P.orphicus*	F	–	*orph*2	Valikardhë	Pure karst soil
ON715917	–	18-111-K95	18D295	–	*P.orphicus*	F	–	*orph*2	Valikardhë	Pure karst soil
ON715918	–	18-111-K25	18D225	–	*P.orphicus*	M	–	*orph*2	Valikardhë	Pure karst soil
ON715919	OP537929	18-111-K78	18D278	–	*P.orphicus*	F	–	*orph*2	Valikardhë	Pure karst soil
ON715923	–	18-124-X103	22A030	–	*P.orphicus*	M	–	*orph*2	Lurë region, NW of Cidhën	Pure karst soil
ON715924	–	18-124-X104	22A031	–	*P.orphicus*	M	–	*orph*2	Lurë region, NW of Cidhën	Pure karst soil
ON715925	OP537930	18-115-K76	18D276	K76	*P.orphicus*	M	n=42	*orph*2	Lurë region, Fushë Lurë	Valley near 2^nd^ mountain, karst soil
ON715926	–	18-115-K66	18D266	–	*P.orphicus*	M	–	*orph*2	Lurë region, Fushë Lurë	Pure karst soil
ON715927	–	18-115-K67	18D266	–	*P.orphicus*	M	–	*orph*2	Lurë region, Fushë Lurë	Pure karst soil
ON715920	–	18-115-X97	22A024	–	*P.orphicus*	M	–	*orph*2	Lurë region, Fushë Lurë	Mixed ophiolite/karst soil
ON715921	–	18-115-X101	22A026	–	*P.orphicus*	M	–	*orph*2	Lurë region, Fushë Lurë	Mixed ophiolite/karst soil
ON715922	–	18-115-X102	22A029	–	*P.orphicus*	F	–	*orph*2	Lurë region, NW of Arras	Mixed ophiolite/karst soil
ON715928	–	18-115-K80	18D280	K80	*P.orphicus*	M	n=42	*orph*2	Lurë region, Fushë Lurë	Mixed ophiolite/karst soil
ON715929	–	18-115-K84	18D284	K84	*P.orphicus*	M	n=42	*orph*2	Lurë region, Fushë Lurë	Mixed ophiolite/karst soil
ON715930	OP537931	18-115-K85	18D285	K85	*P.orphicus*	M	n=42	*orph*2	Lurë region, Fushë Lurë	Mixed ophiolite/karst soil
ON715905	–	17-94-3	17E542	2017–03	P.lurae×orphicus putative **hybrid**	M	n=43, 44	*orph*2	Lurë region, Gurë-Lurë	Nectaring on flowers along road
ON715910	–	18-115-K69	18D269	K69	P.lurae×orphicus putative **hybrid**	M	n=43	*orph*2	Lurë region, Fushë Lurë	Mixed ophiolite/karst soil
ON715921	–	18-115-K83	18D283	K83	P.lurae×orphicus putative **hybrid**	M	n=43	*orph*2	Lurë region, Fushë Lurë	Mixed ophiolite/karst soil
ON715908	–	18-115-K90	18D290	K90	P.lurae×orphicus putative **hybrid**	M	n=43	*orph*2	Lurë region, Fushë Lurë	Mixed ophiolite/karst soil
ON715895	–	18-115-X98	22A025	–	*P.lurae* **sp. nova**	M	–	*orph*2	Lurë region, Fushë Lurë	Mixed ophiolite/karst soil
ON715896	OP537924	18-115-K71	18D271	K71	*P.lurae* **sp. nova**	M	n=41+ trivalent	*aroa3*	Lurë region, Fushë Lurë to Qafa e Lura	Ophiolite soil+mixed, **paratype male**
ON715897	–	18-115-K73	18D273	K73	*P.lurae* **sp. nova**	M	n=42	*aroa3*	Lurë region, Fushë Lurë	Mixed ophiolite/karst soil, **paratype male**
ON715898	OP537925	18-116-K68	18D268	K68	*P.lurae* **sp. nova**	M	n=ca.42	*aroa3*	Lurë region, Qafa e Lura	Ophiolite soil, **paratype male**
ON715899	–	18-116-K70	18D270	–	*P.lurae* **sp. nova**	M	–	*aroa3*	Lurë region, Qafa e Lura	Ophiolite soil, **paratype male**
ON715900	OP537926	18-116-K77	18D277	–	*P.lurae* **sp. nova**	F	–	*aroa3*	Lurë region, Qafa e Lura	Ophiolite soil, **paratype female**
ON715903	OP537927	18-116-K81	18D281	–	*P.lurae* **sp. nova**	M	–	*aroa3*	Lurë region, Qafa e Lura	Ophiolite soil, **paratype male**
ON715901	–	18-116-X100	22A028	K82	*P.lurae* **sp. nova**	M	n=43	*aroa3*	Lurë region, Qafa e Lura	Ophiolite soil, **HOLOTYPE male**
ON715904	–	18-118-K88	18D288	K88	*P.lurae* **sp. nova**	M	n=42	*aroa3*	Lurë region, Pregj Lurë	Ophiolites, **paratype male**
ON715902	–	18-119-K79	18D279	–	*P.lurae* **sp. nova**	M	–	*aroa3*	Lurë region, Pregj Lurë	Ophiolites, **paratype male**

### ﻿Phylogenetic inference

The *COI* analysis involved 60 sequences (19 GenBank sequences and 41 own material). For the *COI* phylogeny, sequences of different length (from 647 to 657 bp) were included into the final dataset alignment. We used Geneious Prime 2019.0.3 (https://www.geneious.com) software to align the sequences and then edited them manually. The final *COI* alignment included 657 sites, with 137 variable sites and 112 parsimony-informative sites. A phylogeny was reconstructed in BEAST v2.5.0 (Bouckaert et al. 2014). Parameters were estimated using two independent runs of 30 million generations each and convergence was checked with TRACER 1.7.1 (Rambaut, 2018). A burn in of 10% was applied. Samples of P. (A.) damon were used to root the tree. Besides, a haplotype network of the *COI* barcode region was created in POPART v1.7 (Leigh & Bryant, 2015) using the TCS method.

Based on Wiemers et al. (2009), *ITS2* secondary structure improves phylogeny estimation of the subgenus Agrodiaetus and thus we combined mitochondrial and nuclear sequences to improve phylogenetic signal, in agreement with Vishnevskaya et al. (2016). This resulted in a concatenated *COI + ITS2* alignment with a total of 1039 bp. Phylogeny on concatenated sequences was reconstructed in BEAST v2.5.0 (Bouckaert et al. 2014) on a subset of Albanian specimen, covering all study sites, and extra sequences mined from GenBank (9 own material and 45 extra sequences). Phylogenetic relationships were inferred using Bayesian Inference (BI), maximum likelihood (ML) and maximum parsimony (MP) analyses. The Bayesian analysis of the concatenated matrix *COI+ITS2* was performed using the program MrBayes 3.2 (Ronquist et al. 2012) with default settings as suggested by Mesquite (Version 3.04. http://mesquiteproject.org): burn-in = 0.25, nst = 6 (GTR + I + G). Two runs of 10 million generations with four chains (one cold and three heated) were performed. The first 25% of each run was discarded as burn-in. The consensus of the obtained trees was visualized using FigTree 1.3.1. (http://tree.bio.ed.ac.uk/software/figtree/). The samples of *P.damon* were used to root the tree. The NJ analysis of the concatenated matrix *COI+ITS2* was performed using the program Mega X (Kumar et al. 2018) and Tamura3-parameter+G as the optimal model (also estimated by Mega X). The samples of *P.damon* were used to root the tree. The standard nonparametric bootstrap (Felsenstein, 1985) (100 replicates) was used to evaluate statistical nodal support of the tree.

### ﻿Analysis of karyotype

Testes were removed within 1 hour after collection and were stored in the 3:1 fixative for several months at +4 °C and then stained with 2% acetic orcein for 30 days at 20 °C. In total we karyotyped a selection of 15 samples representative for the different species and biotopes handled in this paper. We used a two-phase method of chromosome analysis as described in Lukhtanov and Dantchenko (2002). In the first phase, the stained testes were placed into a drop of 40% lactic acid on a slide, the gonad membranes were torn apart using fine needles and intact spermatocysts were removed and transferred into another drop of 40% lactic acid. Intact spermatocysts were studied and photographed. The first phase was most useful for counting the number of chromosome bivalents and multivalents. In the second phase, different stages of chromosome spreading were studied using a slight, gradually growing pressure on the coverslip. The second phase was most useful for studying the chromosome structure and distinguishing between bi- and multivalents. By scaling up the pressure on the coverslip, we were able to manipulate chromosomes, e.g. change their position and orientation on the slide, and consequently to resolve controversial cases of contacting or overlapping bivalents. Haploid chromosome numbers were counted at metaphase I (MI) and/or metaphase II (MII) of meiosis.

Leica DM2500 light microscope equipped with HC PL APO 100×/1,44 Oil CORR CS lens and S1/1.4 oil condenser head was used for bright-field microscopy analysis. Leica lens HC PL APO 100×/1,40 OIL PH3 was used for phase-contrast microscopy analysis.

### ﻿Morphometric measurements of habitus (dorsal wing reflectance)

Wing colour is an important trait for identification of butterflies and a species-specific characteristic (Bálint et al. 2012), an indicator of genetic variation (Wasik et al. 2014), and evidence of a changing population (Hiyama et al. 2012; Kertész et al. 2021). Observing fixed differences in wing colour of butterflies of different population can serve as a reliable tool for species identification (Bálint et al. 2010). Here we used colour measurements of dorsal wings of male *Agrodiaetus* to generate standardized RGB measurements of set specimens. In our set-up a constant light source in a darkened room was used (3 Marbul® suspension light sources of 12 W, 955 lm, 3500 K) in a triangle position at 1 m above the specimen to obtain a reproducible and uniform light source and minimize shades. RGB pictures were made on specimens positioned at an angle of 20° to the equatorial to measure maximum light reflectance generated by wing scale structures. The spectral position of the reflectance maximum of such photonic nanoarchitectures depends on the nanoscale geometric dimensions of the elements building up the nanostructure and was based on earlier experience and method described by Kertész et al. (2021). To obtain a uniform colour zone, the intervein space – which showed most reflectance variability- of the inner postdiscal zone (only between M1 and CU2 cells) was used. Per measurement a circular area of the wing was blurred in Lightroom and the average colour obtained was mapped on a disc. On this uniform colour discs (3 samples per wing), colour measurements were done generating exact RGB and HUE values using the colour picker tool. Averaged values per specimen were used for statistics. Wing colour measurements were taken only from fresh samples (worn specimens and those with minimal damage on fringe were discarded) belonging to the *Polyommatusaroaniensis* species complex collected from different Albanian localities, with habitats harbouring ophiolitic, karst and mixed substrates, as detailed in Suppl. material 1.

### ﻿Statistical analysis of wing morphometrics

NMDS plots based on 27 specimens were obtained using the Adonis script (Vegan package) in R (Oksanen et al. 2016). Ellipses indicating 95% confidence intervals representing species identifications based on *COI* results (*orph2* and *aroa3*) and karyotype (*orphicus*, *lurae*), obtaining the categories ‘orphicus’, ‘lurae’, and ‘hybrid’ (as a few specimens showed mitochondrial-karyotype discordance). In the analysis, substrate type was also integrated as a factor, with three categories ‘ophiolite’, ‘karst’, and ‘mixed’.

Posterior statistics was done running a permutational multivariate analysis of variance, using distance matrices with the Adonis call (Vegan package) in R (Oksanen et al. 2016).

## ﻿Results

### ﻿Biotopes and Albanian samples

**Figure 1.**
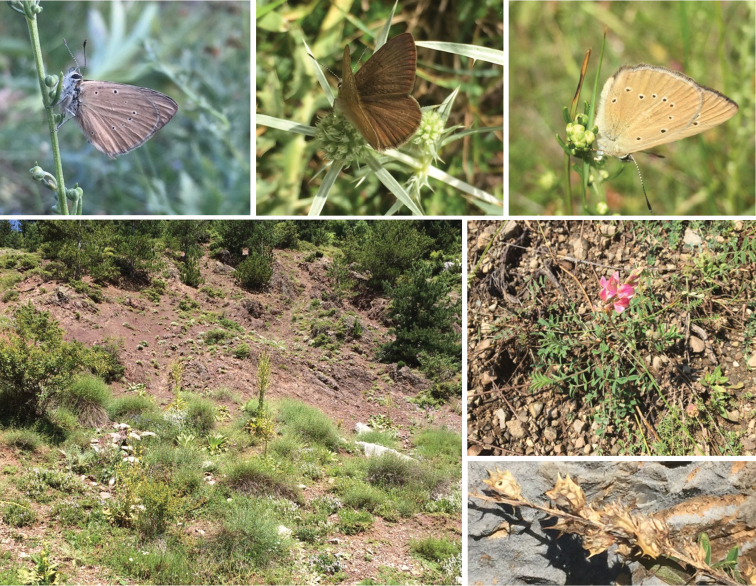
Biotope and specimens in their natural biotopes at the Lurë region **a***P.lurae* sp. nov uns. (sample code 18-115-K71), Albania, Dibër prov., Lurë region, 24.VII.2018 in its biotope **b***P.lurae* sp. nov ups., same specimen **c** Albanian *P.orphicus* (sample code 18-124-X103) in its biotope on Karst substrate, Dibër prov., Lurë region, 25.VII.2018, NW of Cidhën village **d** typical biotope of *P.lurae* with dark ophiolitic soil substrate **e, f***Onobrychisalba*, host plant for Polyommatus (Agrodiaetus) sp., growing in ophiolitic soil substrate.

In total, 251 *Agrodiaetus* samples belonging to the *P.aroaniensis* and *P.ripartii* species complexes were analysed. Specimens were collected from 94 Albanian different sites visited and distributed from Southern up to North-eastern Albania. Only in a minor part of the sites (7/94 sites), specimens were identified as belonging to the *P.aroaniensis* species complex, mostly in provinces Dibër and Korçë. Specimens with clear *P.ripartii* traits – based on habitus descriptions given by Tshikolovets (2011) and Vishnevskaya et al. (2016) – were excluded from the analyses, after some voucher samples were barcoded to confirm our identifications (data not shown). Especially the study sites in the Dibër province, Lurë region, attracted our attention because many individuals displayed a remarkably dark habitus and mostly were completely lacking the white stripe on the hindwing underside (Fig. 1a, b). Their biotope was atypical, with a dark ophiolitic substrate (Fig. 1d). Here, in the vicinity, *Onobrychisalba*, a known foodplant of different *Agrodiaetus* species, was found growing (Fig. 1e, f). Besides, in another region, near Valikardhë village, a second cluster of study sites potentially harbouring *orphicus/aroaniensis* populations (Fig. 1c) was identified, although the habitat soil type was visibly paler and consisted of typical karst (chalk) substrate.

### ﻿Phylogenetic reconstruction

**Figure 2.**
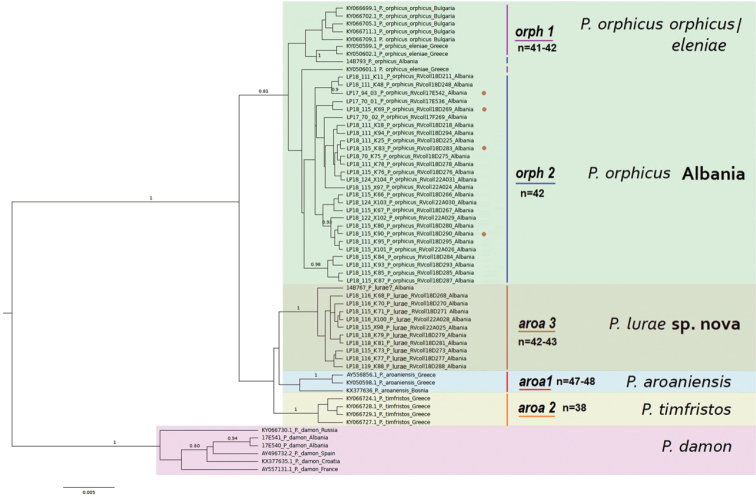
Bayesian inference tree based on 658-bp of the gene *COI* for the entire *Polyommatusaroaniensis* species complex. Node posterior probabilities (0.80 and higher) are written on the branches. The scale bar indicates the length of 0.005 substitutions/site. Main clades are highlighted and *COI* haplogroups and karyotype numbers indicated: *P.orphicusorphicus/eleniae* (*orph1*, n=41–42), *P.orphicus* Albania (*orph2*, n =42), *P.aroaniensis* (*aroa1*, n=47–48), *P.timfristos* (*aroa2*, n=38) and *P.lurae* sp. nov (*aroa3*, n=42–43). Brown dots next to specimens within the *orph2* clade indicate *P.lurae × P.orphicus* potential hybrids (n=43–44) found in the contact zone or dispersing.

The phylogeny obtained by Bayesian inference based on 658-bp of the gene *COI* (Fig. 2) recovered the *P.orphicus* and *P.aroaniensis* species groups as distinct lineages (although with moderate support), in agreement with previous studies (Wiemers 2003; Lukhtanov et al. 2005; Vila et al. 2010; Lukhtanov et al. 2015; Vishnevskaya et al. 2016). *Polyommatusorphicusorphicus* and *P.orphicuseleniae* formed together a paraphyletic cluster (orph 1), while most of the Albanian *P.orphicus* specimens formed a sister group (orph 2), albeit node supports were low. The *P.aroaniensiss.l.* clade showed three highly-supported clades (posterior probability = 1) corresponding to *P.aroaniensis* (aroa 1), *P.timfristos* (aroa 2), and another one (aroa 3) exclusively composed by Albanian specimens, hereunder described as *P.lurae* sp. nov.

**Figure 3.**
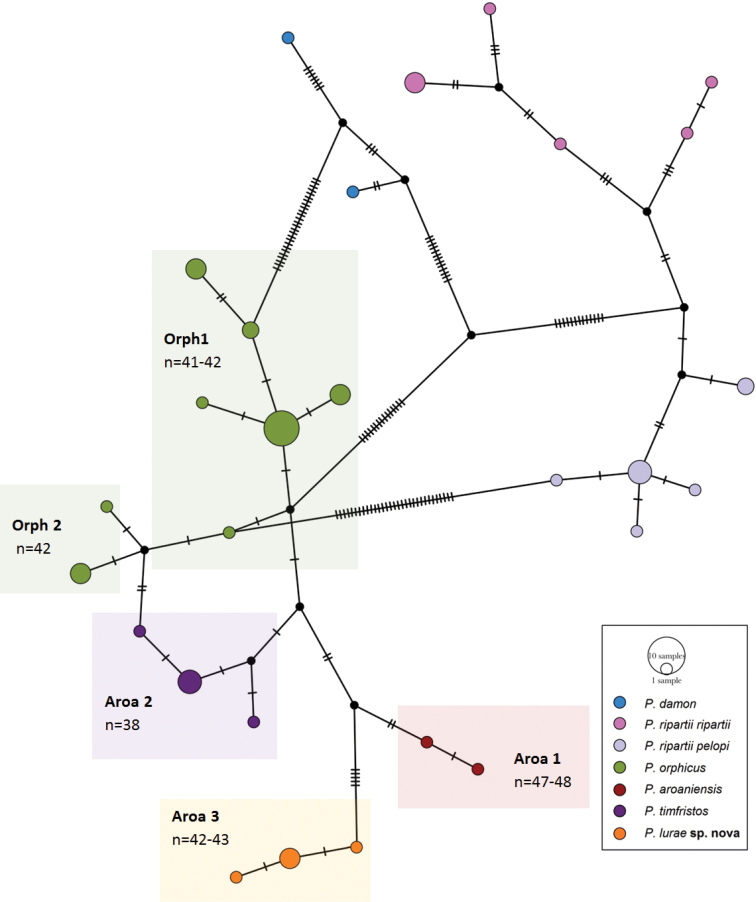
Haplotype network of the *P.aroaniensis* species group in relation to *P.damon* and *P.ripartii*. Coloured circles represent different taxa, as indicated in the legend; coloured boxes delimitate the haplogroups discussed in the text. Each line segment represents a mutation step, and black small circles represent “missing” haplotypes.

To construct the haplotype network, we used 54 specimens that were collapsed in 28 haplotypes representing 7 haplogroups (Fig. 3) with *P.damon* as outgroup: 3 haplogroups for the *P.ripartiispecies* complex (including *P.ripartiiripartii* and *P.ripartiipelopi*), 2 for *P.orphicus* (orph 1, and orph 2 representing the Albanian lineage) and 3 for the *P.aroaniensiss. l.* clade including *P.timfristos* (aroa 2), *P.aroaniensis* (aroa 1) and *P.lurae* sp. nov (aroa 3). The latter haplogroup is clearly distinct from related taxa as can be seen in the number of nucleotide substitutions between them.

The phylogenies based on the concatenated COI+ITS2 sequences (54 specimens, same as the haplotype network) and using the BI and NJ methods are given in Suppl. material 2. The NJ analysis of the concatenated matrix (using Tamura3+G as the best model) (Suppl. material 2, Fig. 1) revealed *lurae* as a highly supported, differentiated lineage with the most basal position within the *entire aroaniensis* species complex. However, this basal position had a low support. The BI analysis of the concatenated matrix (using default settings) (Suppl. material 2, Fig. 2) revealed *lurae* as a highly supported lineage, sister to *aroaniensis* (but the support for this sister relationship was very low). Generally, the concatenated alignments revealed the same topology as in the case of the COI tree, with very good support of P. *lurae* sp. nov as a well differentiated lineage, and putative sister to *P.aroaniensis*, although with lower support.

In all cases using different phylogenetic reconstruction models, and based on COI or concatenated *COI+ITS2* sequences, *P.lurae* sp. nov formed a monophyletic, well differentiated clade with very good support.

### ﻿Karyotyping

#### *Polyommatusorphicus* from Albania (populations in which the only o*rph2* haplogroup is present)

**Figure 4.**
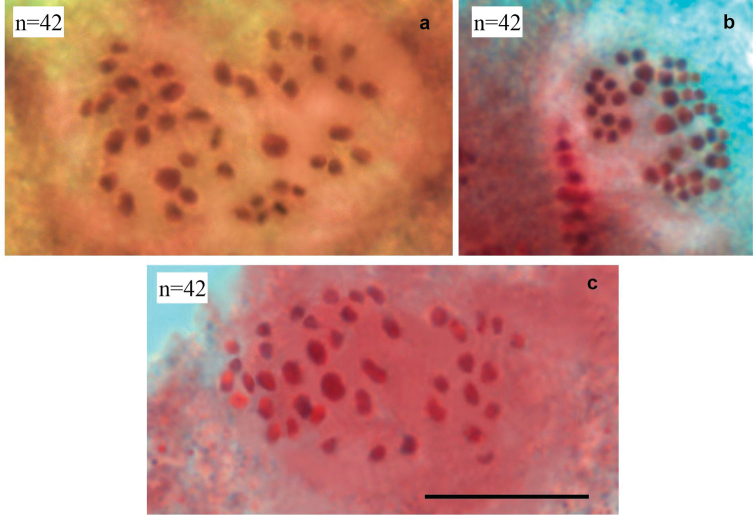
Karyotypes of *Polyommatusorphicus* from Albania **a** sample K75, MI, n=42 **b** sample K76, MII, n=42 **c** sample K80, MI, n=42. Scale bar: 10 μm.

In five studied samples (K75, K76, K80, K84, K85) the number of countable elements was found to be n=42 at MI and MII cells. Bivalents at MI and univalents at MII were fairly well differentiated with respect to their size; however, it was difficult to subdivide them objectively into size groups because the sizes of the elements decrease more or less linearly (Fig. 4).

#### *Polyommatuslurae* sp. nov from Albania (populations in which the only *aroa3* haplogroup is present)

**Figure 5.**
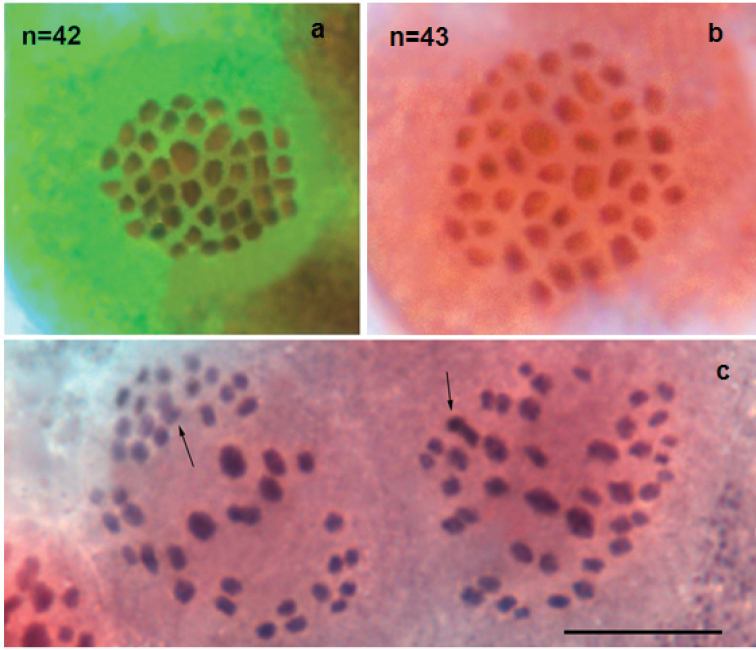
Karyotypes of *Polyommatuslurae* sp. nov from Albania **a** sample K73, MII, n=42 **b** sample K82, MII, n=43 **c** sample K71, two MI cells, each cell displays 41 bivalents and one possible trivalent (arrow). Scale bar: 10 µm.

In five studied samples (K68, K71, K73, K81, K88), two different haploid chromosome numbers (n=42 and n=43) were observed at MI and MII cells of the 14 specimens studied. This variation was most likely caused by polymorphism for one chromosome fusion/fission. This polymorphism resulted in three types of MI karyotype: n=42 (homozygous for chromosomal fusion/fission, one pair of fused chromosomes; 2n=84), n=43 (homozygous for chromosomal fusion/fission, two pairs of unfused chromosomes; 2n=86) and n=42 (heterozygous for chromosomal fusion/fission, resulting in 41 bivalents and one trivalent; 2n=85). Bivalents at MI and univalents at MII were fairly well differentiated with respect to their size; however, it was difficult to subdivide them objectively into size groups because the sizes of the elements decrease more or less linearly (Fig. 5).

#### Contact zone with *Polyommatuslurae × P.orphicus* potential hybrids (area where both o*rph2* and a*roa3* haplogroups are present)

**Figure 6.**
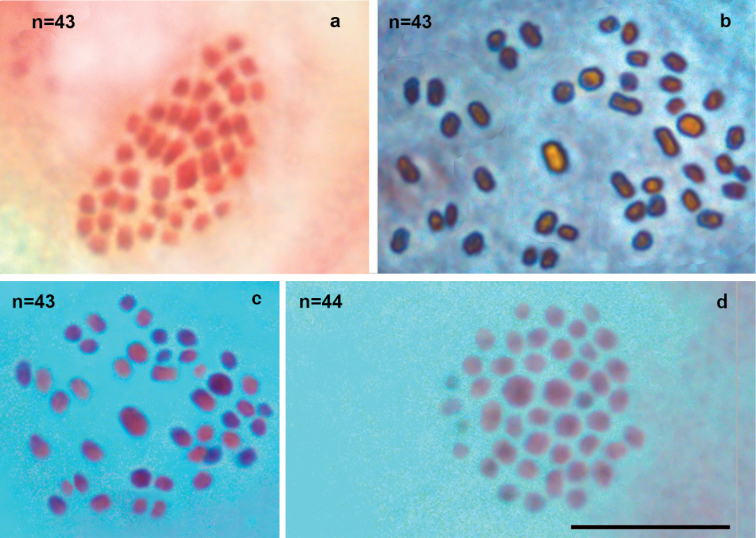
Karyotype of two putative hybrid specimens from the contact zone between *P.orphicus* and *P.lurae* sp. nov, where both orph2 and aroa3 haplogroups are present **a** sample K69, MII, n=43 **b–d** sample 17-094-3: **b**MI, n=43 (phase-contrast) **c**MI, n=43; **d**MI, n=44. Scale bar: 10 μm.

The contact zone between the two species was defined as the area where both *orph2* and *aroa3* haplogroups were found to coexist, and coincided with a mixed ophiolite/karst substrate. In this area, in addition to specimens typical to either species, a number of countable elements of n=43 at MI and MII cells was found for three samples with a mitochondrial haplogroup *orph2* (samples K69, K83, and K90). Bivalents at MI and univalents at MII were fairly well differentiated with respect to their size; however, it was difficult to subdivide them objectively into size groups because the sizes of the elements decrease more or less linearly (Fig. 6). In the sample 17E542 (also haplogroup *orph2*) a karyotype n=44 was also observed at the MI stage, along with n=43, most likely due to intraindividual chromosome fragmentation or a single-chromosome disjunction. This sample was found outside the contact zone nectaring on the flowers along a road and could be a dispersive specimen.

### ﻿Distribution of species within *P.aroaniensis* complex in the Balkans

**Figure 7.**
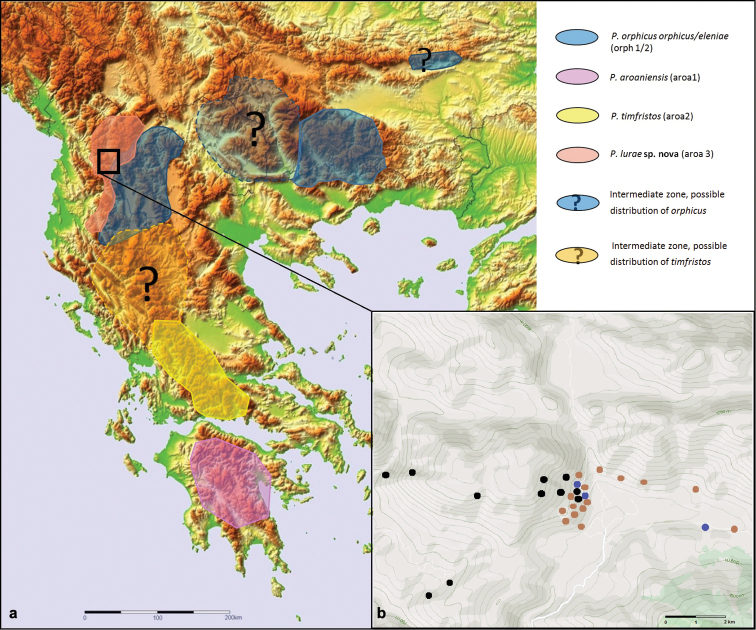
**a** distribution map based on literature and predictions of the anomalous blues within the *P.aroaniensis* species complex in the Balkan peninsula; colours correspond to the species in the legend; box is indicating the Lurë region in Albania **b** detailed map of the Lurë region with observations of *P.lurae* sp. nov (black dots) on dark ophiolites, *P.orphicus* (brown dots) and putative hybrids (blue dots) in the contact zone (blue dots) and a dispersive specimen outside suitable biotope (paler blue dot).

A distribution map based on current literature and predictions of the anomalous blues within the *P.aroaniensis* species complex in the Balkan peninsula is shown in Fig. 7a and a detailed map of the specimens found in Lurë region in Fig. 7b While several populations from Central and Northern Greece, as well as from other countries of the Balkan Peninsula were formerly identified as *P.aroaniensis*, based on Vishnevskaya et al. (2016)*P.aroaniensis* is only found in Southern Greece (Peloponnese). The sister taxon *P.timfristos*, of which it was then separated is currently only known from Mt. Timfristos and Mt. Parnassos in Central Greece. However, there is still a missing gap existing between *P.lurae* sp. nov and *P.timfristos* in Central to Northern Greece and it is not impossible that the latter can be found further northwards in suitable karst biotopes. For *P.orphicus*, the taxon is generally found in the northern parts of the Balkans (confirmed in Northern Greece and Bulgaria, and in North Macedonia). Here, we described also new populations of *P.orphicus* in Albania, and the Lurë area is the westernmost distribution of the species in the Balkans. Yet, between the Albanian populations and the former mentioned there still exist an intermediate gap of at least 500 km with insufficient distribution knowledge. Finally, in the light of the data obtained in this paper, the (possible) occurrence of *P.aroaniensis* s.l. in Kosovo, Northern Greece, North Macedonia needs to be further investigated as they could harbour unknown *P.orphicus* populations, and probably also populations of *P.lurae* sp. nov because biotopes with ophiolites exist there.

### ﻿Morphometrics of male wings

**Figure 8.**
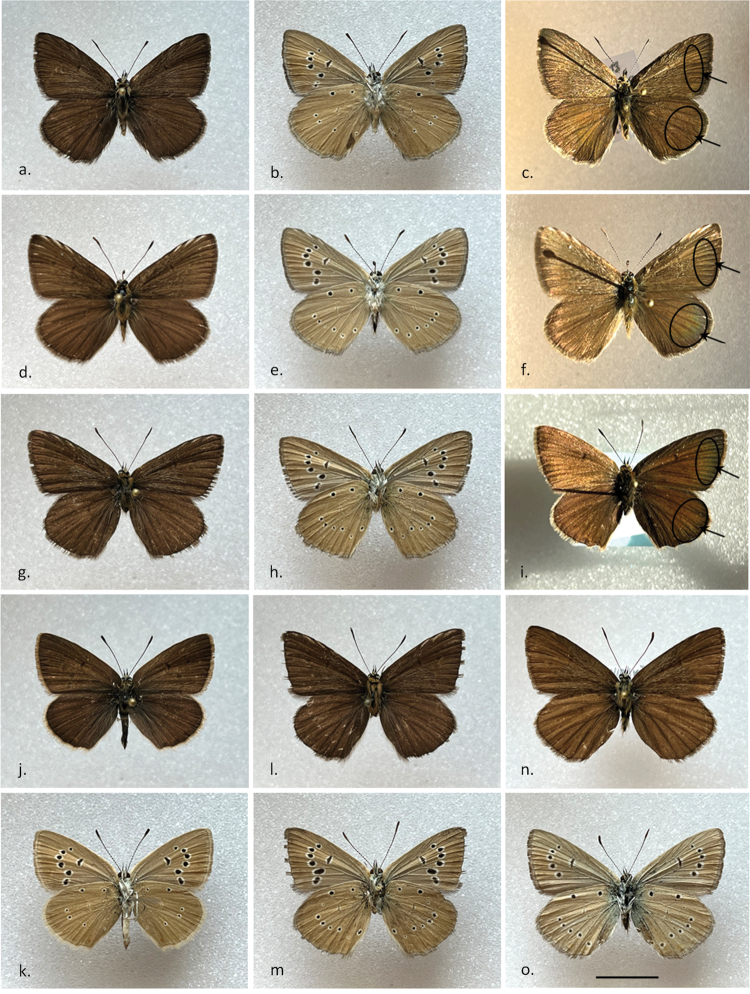
The colouration and wing pattern of *P.lurae* sp. nov, *P.orphicus* and *P.ripartii*. The letters correspond to the following species (and voucher sample codes as listed in Table 1): **a–c***P.lurae* sp. nov **HT male** (18-115-K71) **d, f***P.lurae* × *P.orphicus* putative hybrid (17-94-3) **g, i***P.orphicus* (18-115-K76) **j, k PT female** (18-116-K77) **l, m***P.lurae***PT** (18-116-X100) **n–o***P.ripartii* (same collecting data as 18-115-K76). Pictures of **c, f, i** were taken in natural sunlight but with same position as morphometric analysis (see in M&M section); Notice the differences in reflectance on dorsal wings in the postdiscal zone which is circled and indicated by arrow. Scale bar: 10 mm.

The upperside wing colour is one of the main characteristic features of the anomalous blue butterflies. Vishnevskaya et al. (2016) used some external traits of the wing underside for differentiating between forms (types) of Balkan specimens under the “brown” complex, which we here reanalyse in view of Albanian taxa, here described for the first time, and identified under cryptic taxa *P.ripartii*, *P.orphicus* and *P.lurae*:

1. “ *Polyommatusripartii* type”: hindwing underside with well-developed white streak, spots are small or medium-sized, marginal marking is reduced. According to Vishnevskaya et al. (2016) this type is found in different species including *P.admetus*, *P.timfristos*, *P.orphicus*, and *P.ripartiipelopi* (plates of male specimens shown in Vishnevskaya et al. (2016), which we confirm for the latter two species analysed. This type was never found in *P.lurae* specimens (data not shown).
2. “ *Polyommatusaroaniensis* type”: the white streak on the hindwing underside demon­strates different level of reduction. This type is found in *P.aroaniensis* s.s., *P.lurae* sp. nov (Fig. 8m). *P.timfristos*, *P. orphicus orphicus/eleniae* and Albanian *P.orphicus* (Fig. 8h). It is also found in the population of *P.ripartii* from the Crimea (Vila et al. 2010) and Croatia (Lovrenčić et al. 2016), but according Vishnevskaya et al. (2016) very rare in the Balkan peninsula while we also found some *ripartii* specimens with great reduction of the white stripe (example in Fig. 8o).
3. *Polyommatusorphicus* type: forewing underside with clear white postdiscal streak between discal spot and submarginal marking, white streak on hindwing under­side is prominent, often with an additional small white streak. According to Vishnevskaya et al. (2016) this type is common in *P.orphicusorphicus* while mentioning that its most characteristic feature (the white postdiscal streak between discal spot and submarginal marking on the forewing underside) can be found in other species, e.g. *P.aroaniensis.* Only about one third of the Albanian specimens of *orphicus* showed the additional streak (data not shown), while it was never present in specimens of *P.lurae*.
4. *Polyommatuslurae* sp. nov type: forewing underside with no white postdiscal streak between discal spot and submarginal marking, white streak on hindwing under­side is minimal and mostly completely lacking or invisible (Fig. 8b). This type is also found in a minor amount of Albanian *P.orphicus* (Fig. 8h). Based on pictures of the type series, also some *P.aroaniensis* specimens from Greece harbour this trait (Brown 1976).

**Figure 9.**
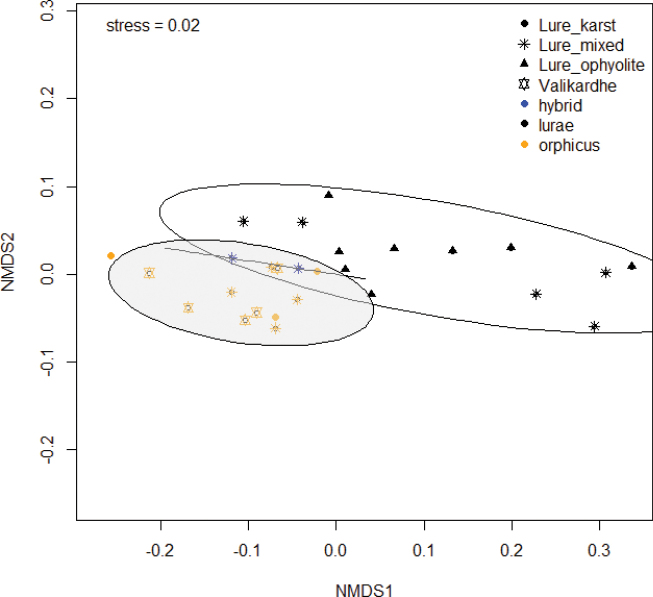
NMDS plot representing the morphometric analysis of dorsal male wings reflectance measurements of *P.orphicus* (orange dots) and *P.lurae* sp. nov (black dots). Stress = 0.02; specimens indicated in blue are potential *P.lurae* × *P.orphicus* hybrids collected in the contact zone and showed intermediate wing reflectance. Different symbols represent collection sites of three studied habitat with different substrate types (ophiolitic, karst, mixed). Ellipses represent 95% confidence intervals of specimen groups ‘orphicus’, ‘lurae’ and ‘hybrid’.

We also analysed light reflection of male wings based on standardized colour measurements (RGB and HSV values) for 27 specimens. We focused on the species *P.orphicus* and *P.lurae*, and also included the few potential hybrid specimens (based on the atypical combination of mitochondrial haplogroup and karyotype results and always collected at the contact zone in the Lurë region. NMDS plots (Fig. 9) showed that wing reflection measurements matched significantly the phylogenetic clades *aroa3* and *orph2* (Df= 2, F=4.11, P=0.030). Specimens identified as *P.orphicus* generally showed a measurable reflectance on the postdiscal band of forewing (and hindwing) (Fig. 8h), while this trait is absent in *P.lurae* sp. nov (Fig. 8c). Interestingly, the two potential hybrids analysed showed an intermediate position in wing colour reflectance, falling in the overlapping area of the two species, as visualized by blue dots in Fig. 9. The intermediate reflectance is also noticeable on the putative hybrid specimen depicted in Fig. 8f.

Next to this, a link with the soil substrate was tested and statistical analysis revealed that both species *P.lurae* and *P.orphicus* could significantly be linked with their locations harbouring typical soil substrates, i.e. dark ophiolitic versus light karts soils, respectively (Df= 3, F=4.39, P=0.014).

### ﻿Taxonomy

The results showed a consensus between morphometrics, mitochondrial DNA and karyotype, in delineating three clades under the *P.aroaniensis* species complex. Two of them are generally accepted as species: *P.aroaniensis* and *P.timfristos* (Wiemers et al. 2018). Given that the genetic, morphological and karyotypic differentiation of the third clade is comparable to that between the other two species, we describe the Albanian population as a new species belonging to the *P.aroaniensis* s.l. species complex.

#### ﻿ Polyommatus lurae

Taxon classificationAnimaliaLepidopteraLycaenidae

Parmentier, Vila et Lukhtanov sp. nov

676BCA54-343E-549D-A297-41A8EF05B05F

https://zoobank.org/A561FDF8-DA47-4814-A976-C57A7F260386

##### Description.

Typical dark ground colour of both veins and intervein space of dorsal wing sides. A character that appears useful for separation of *P.orphicus* and *P.lurae* sp. nov is the brighter yellow-greenish reflection of the former which is generally lacking in the newly described taxon. However, worn individuals of the two taxa may be indistinguishable externally and also from *P.ripartii*, which is found sympatrically in all locations studied. While Misja (2005) reports *P.admetus* from the same Lurë region, we never found *P.admetus* in sympatry with the new taxon in all Lurë locations surveyed. While the latter observation may be based on a wrong identification of the newly described taxon, also lacking white stripes on the hindwing underside, *P.admetus* is easily separated from *P.lurae*, especially because of the strongly marked underside in *P.admetus*, with a double row of small dots in the submarginal zone of underside wings, which has never been observed nor reported in literature in the taxa of the *P.aroaniensis* species complex, including *P.lurae*.

##### Holotype.

(Fig. 8a–c) Male, field code specimen 18-116-X100, *COI* barcode number RVcoll22A028 (DNA extraction in RVcoll, Barcelona, Spain), GenBank accession number ON715901. Locus typicus: Albania, Dibër prov., Lurë region, Mountain ridge with ophiolitic soil substrate North of Cidhën near Fushë Lurë, 1250 m., 24.VII.2018, L. Parmentier leg. et coll. Holotype in LPA collection, Zulte, Belgium

##### Paratypes.

Nine males and one female were studied in depth, with field codes of voucher specimen in LP collection (RVcoll number/ GenBank accession numbers of barcodes): LP18-115-K71 (RVcoll18D271/ ON715896), LP18-115-K73 (RVcoll18D273/ON715897), LP18-116-K68 (RVcoll18D268/ ON715898), LP18-116-K70 (RVcoll18D270/ ON715899), LP18-116-K77 (RVcoll18D277/ ON715900), LP18-116-K81 (RVcoll18D281/ ON715903), LP18-116-X100 (RVcoll22A028/ON715903), LP18-115-X98 (RVcoll22A025/ON715895), all North of Cidhën near Fushë Lurë, 1050–1600m. 23–24.VII.2018; LP18-118-K88 (RVcoll18D288/ ON715904), LP18-119-K79 (RVcoll18D279/ ON715902) Lurë region, Pregj Lurë 24.VII.2018. Additional material: 15 males, 5 females, same localities, collection dates 23- 24.VII.2018. All paratypes have red labels indicating *P.lurae* sp. nov, name of authors, signature of first author and exact localities.

##### Karyotype.

The haploid chromosome number *P.lurae* sp. nov is determined as n=42–43 (Fig. 4).

##### *COI* barcode sequence of the holotype.

657 base pairs: AACATTATATTTTATTTTTGGTATTTGAGCAGGAATAGTAGGAACATCTCTAAGAATTTTAATTCGTATGGAATTAAGAACTCCTGGATCCTTAATTGGAAATGATCAAATTTATAATACTATTGTTACAGCTCATGCATTTATTATAATTTTTTTTATGGTTATACCTATTATAATTGGAGGATTTGGTAACTGATTAGTTCCCTTAATATTAGGAGCACCTGATATAGCCTTTCCCCGATTAAATAATATGAGATTTTGATTATTACCACCATCATTAATACTACTAATTTCTAGAAGAATTGTAGAAAATGGTGCAGGAACAGGATGAACAGTTTACCCCCCACTTTCATCAAATATTGCACATAGAGGATCATCTGTAGATTTAGCAATTTTCTCTCTTCATTTAGCAGGAATTTCTTCAATTTTAGGAGCAATTAATTTTATTACAACTATCATTAATATACGAGTAAATAATTTATCTTTTGATCAAATATCATTATTTATTTGAGCAGTGGGAATTACAGCATTATTATTACTTTTATCATTGCCTGTATTAGCTGGAGCAATTACCATATTACTAACAGATCGAAACCTTAATACCTCATTCTTTGACCCAGCTGGTGGAGGAGATCCAATTTTATATCAACATTTATTT.

##### Description.

**Males.** (Fig. 8c, l, m). Forewing length 15.8–17.9 mm. ***Upperside***: ground colour completely dark chocolate brown. Discoidal, submarginal and antemarginal markings absent on both fore- and hindwings. Veins poorly contrasting. Forewings with a developed sex brand and dark scale tuft. Fringe grayish brown. ***Underside***: ground colour yellow-brown with ochreous to reddish coffee-milk tint. Minimal greenish blue basal suffusion. One basal black spot is present only on hindwings. Discoidal black spot is present on the forewings, but can be slightly seen on the hindwings (absent or vestigial). Postdiscal black ocelli most prominent on forewings; when present encircled by a whitish border. Postdiscal black ocelli on the hindwing small and sometimes lacking. Submarginal and antemarginal mark­ing is absent on the forewings, and absent or vestigial on the hindwings. White streak on hindwings generally absent or very faint. Only rarely, the white streak is vestigial; no single specimen was observed with an additional short streak between postdiscal and submarginal areas of the wing, straight under the main white streak. Fringe brown, slightly darker than the underside ground colour.

**Figure 10.**
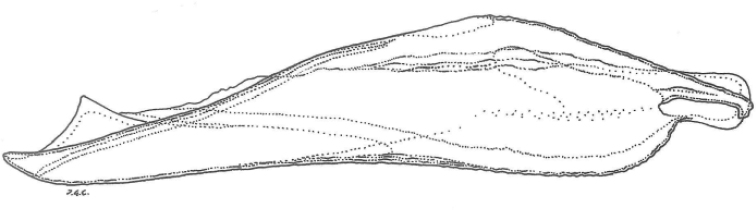
Valva of male genitalia of *P.lurae* sp. nov (G. Coutsis prep. 2018)

***Male genitalia*.** The valva of the male genitalia of *P.lurae* sp. nov is depicted in Fig. 10. Male valves have a structure typical for other species of the subge­nus *Agrodiaetus* (Coutsis (1986), Coutsis, pers. comm.). According to Kolev (2005) who studied the morphometry of the male genitalia of *P.orphicus* no overlap with *P.ripartii* was observed. As male genitalia within the *P.aroaniensis* species group do not significantly differ from each other, those from *P.lurae* may follow the same trend, but no additional analyses nor measurements have been performed.

**Females.** Forewing length 15.8–17.5 mm. ***Upperside***: ground colour as in males, but lighter dark brown and without sex brand and scaletuft. Fringe greyish brown. ***Underside***: ground colour and general design as in males but fringes lighter-coloured. Greenish blue basal suffusion almost invisible. White streak on hindwing underside mostly absent (Fig. 7j, k). If present, it demonstrates a variable level of reduction.

##### Life history.

*Polyommatuslurae* inhabits xerothermic and xeromontane ophiolitic habitats. While in some of the localities the soil can be mixed with a minor degree of a calcareous component, *P.lurae* was never found at pure calcareous biotopes. Indeed, at such localities only *P.orphicus* was found, together with *P.ripartii*, which is in agreement with the original description of these species (Kolev, 2005). The vegetation of the type locality is sparse and dominated by low-growing grasses and flowering plants identified as *Artemisiaalba* Turra and *Saturejamontana* Linnaeus. Besides, other xerophilous species were observed, including scattered *Juniperus* bushes and low *Pinusnigra* trees (Fig. 1d). In all known localities *P.lurae* is syntopic with *P.ripartii*, a species widespread in the Balkans, although especially abundant in calcareous habitats (pers. obs. L. Parmentier).

##### Distribution and biotope.

The three known localities of *P.lurae* (including the type locality) are situated in the Lurë region, in the vicinity of the National Park (Parku Kombëtar Lurë-Mali i Dejës), North of the village Cidhën, along a North-Southern orientated mountain ridge and gorge at altitudes between 950 and 1.600 m (Fig. 9a). The habitats are all situated within ophiolitic soil substrates (in some localities these substrates are slightly intermixed with a minor amount of lighter karst substrate), which are not rare in some parts of Albania. In these typical ophiolitic soil substrates the presumed host plants of the genus *Onobrychis* were observed (Fig. 1e, f). However, there are as yet no observations regarding the first stages of this taxon and the larval host plant is unconfirmed.

The aforementioned ophiolitic substrates can be found in a discontinuous range from Southern Albania (Provinces Korcë, Qukës) up to the Northern part of the country (provinces Dibër, Kukës). Within Europe these rather rare substrates are present mostly in Albania, while neighbouring countries of North Macedonia and Kosovo contain them to a minor degree. Thus, it is not impossible that the species is also present in other ophiolitic habitats where the presumed host plant is growing. Collection material from another locality in Voskopojë (Korçë prov.), situated more South, also harbouring typical dark ophiolitic soils was studied. In this locality, a single specimen (RVcoll14B767) genetically attributable to *P.lurae* was found by Sylvain Cuvelier and Morten Mølgaard, but it is not included in the type series because of the lack of karyological data and morphometrics. Additional specimens from this locality could not be found even after thorough explorations in 2018 and 2022, while only *P.ripartii* could be confirmed.

##### Differential diagnosis.

From nominotypical *P.orphicus* the new taxon is generally distinguished by the strong reduction of a white postdiscal streak on the forewing underside, a darker colour of the upperside and underside wing, lack of wing reflectance, and less contrasting veins on the upperside. Its karyotype is different by at least one fixed chromosome fission (n=41–42) and its *COI* barcode. From *P.aroaniensis*, which is the most similar taxon externally, fresh individuals of the new taxon are distinguished by the constant presence of a typical dark ground colour of both veins and intervein space of dorsal wing sides and a generally darker colour of the upperside and underside wing (while in *aroaniensis* a warm reddish brown colour is typical). However, worn individuals may be indistinguishable externally, while they still can be identified by karyotype (n=48) and by the *COI* barcode. In the case of *P.lurae*, its dark habitus is linked to its typical environment with dark ophiolites, while the taxa *P.orphicus* and *P.aroaniensis* are generally found in biotopes with paler karst soil substrate. From the sympatric and syntopic *P.ripartii*, the new taxon is more easily distinguished by the absence of a white postdiscal streak on the forewing underside and, on average, a more reduced appearance of postdiscal spots, and on the upperside the veins are less pronounced and of a similar tone than the paler ground colour. This may be useful for discriminating even slightly worn individuals of the two taxa, while worn individuals are mostly indistinguishable externally. Yet, its karyotype (n=90) and *COI* barcode are strongly different. *P.admetus* has not been observed on the same biotopes and thus the new taxon could be separated geographically. Besides, *P.admetus* has a very distinct appearance by especially its strongly marked underside (with a double row of small dots on the marginal to submarginal zone of the underside hindwings, a trait that is lacking in the aforementioned species.

##### Etymology.

Derivatio nominis.

The adjective *lurae* has two meanings: “ascribed to Lurë” and “surviving attacks of congeners”. First, the species name is deducted from the Albanian “Lurë region, where the type locality lies, and referring to the old village Lurë e Vjetër situated in central-Eastern Albania (Dibër province). The name alludes to the fascinating history of the old Lurë village: during the Ottoman war, the village was asked 300 women by the enemies. Armed men, disguised with the *duvak*, the traditional red bridal veil, were sent instead on horseback to the Ottoman camp. As a result, the Ottomans were taken by surprise and the Lura tribe eventually won the battle. Also, this second meaning seems adequate for the taxon *lurae*: this species likely experienced periods of close contact with congener species more largely distributed in the Balkans, as is the case at present, but nevertheless has been able to avoid complete admixture and still survives in its unique ophiolitic biotope.

## ﻿Discussion

### ﻿Colour morphometrics, a new method for identification of cryptic *Agrodiaetus* taxa

The use of standardized light reflectance measurements to discriminate between species is a recent method used for identification (Bálint et al. 2010; Bálint et al. 2012; Wasik et al. 2014). Other morphological traits such as underside markings and prominence of the white stripe are useful, but they are not discriminative enough to unambiguously distinguish between the taxa here studied. Most of *P.lurae* specimens have a dark ground colour in the underside of the wings, and no or a very faint white stripe. However, one specimen identified as *lurae* by mitochondrial DNA, karyotype and morphometrics (colour reflectance of dorsal wing colour was typical) showed a more pronounced white stripe. Here we only used RGB an HSV values in the analysis, but more sophisticated measurements to generate full reflectance spectra and SEM graphs may be more powerful to discriminate between these taxa. Such analyses could also shed light on the physical structures that generate the typical dark colour of *P.lurae*, compared to the greenish reflectance in *P.orphicus* specimens, as has been demonstrated in other species of *Polyommatus* (Bálint et al. 2012). Besides, morphometrics on preimaginal stages could also be potentially interesting. Almost no information is available on this aspect, while recent findings showed that differences in larval morphology and in larval host plant preferences may be key in resolving the taxonomy of cryptic species (Hernández-Roldán et al. 2016; Hinojosa et al. 2022).

### ﻿Karyotyping and difference with related taxa

The karyotype of *P.orphicus* was studied previously (Kolev 2005; Vishnevskaya et al. 2016) from localities in Northern Greece and Bulgaria. Two different haploid chromosome numbers (n=41 and n=42) were found to be present in these populations. The variation in chromosome numbers in these populations was explained by polymorphism for one chromosome fusion/fission. This polymorphism resulted in three types of MI karyotype: n=41 (homozygous for chromosomal fusion/fission, one pair of fused chromosomes, 2n=82), n=42 (homozygous for chromosomal fusion/fission, two pairs of unfused chromosomes; 2n=84) and n=41 (heterozygous for chromosomal fusion/fission, 40 bivalents and one trivalent; 2n=83) (Vishnevskaya et al. 2016).

The *P.orphicus* karyotype found by us in Albania (n=42) fits into the previously described variability. At the same time, it can be assumed that in the Albanian population there is a tendency to fixation of the chromosome number n=42, although the studied data are still insufficient to consider this proved.

**Figure 11.**
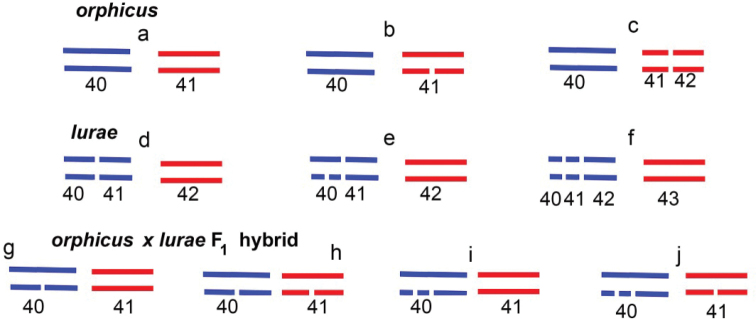
Scheme showing variation in number of chromosomes (lines) and visible elements (=bivalents+multivalents) in MI meiosis in *P.orphicus*, *P.lurae* and their putative F1 hybrids. **a***P.orphicus*, homozygous for chromosomal fusion/fission, one pair of fused chromosomes, 41 visible elements **b***P.orphicus*, heterozygous for chromosomal fusion/fission, 40 bivalents and one trivalent; 41 visible elements **c***P.orphicus*, n=42 (homozygous for chromosomal fusion/fission, two pairs of unfused chromosomes; 42 visible elements **d***P.lurae*, homozygous for chromosomal fusion/fission, one pair of fused chromosomes, 42 visible elements **e***P.lurae*, heterozygous for chromosomal fusion/fission, 41 bivalents and one trivalent; 42 visible elements **f***P.lurae*, homozygous for chromosomal fusion/fission, two pairs of unfused chromosomes; 43 visible elements **g–j** different variants of F1 hybrids. These variants include tri- and quadrivalents; however, the number of visible elements in MI remains 41.

The taxon we describe as *P.lurae* sp. nov also exhibits intrapopulation variability in chromosome numbers (n=42, n=43; estimated diploid numbers are 2n=84, 2n=85, 2n=86) due to polymorphism for one chromosome fusion/fission, but most likely in another chromosome pair. Thus, despite chromosome polymorphism in each of the taxa *P.orphicus* and *P.lurae*, they have, most likely, a fixed difference in one chromosome pair (Fig. 11).

In the contact zone in Albania, both mitochondrial haplogroups *orph2* and *aroa3* occur together. It can be assumed that they arose as a result of hybridization, which is confirmed by the intermediate nature of the colour of the wings.

In the case of hybridization, if contacting taxa have postzygotic reproductive isolation, then hybrid individuals should represent only F1 hybrids (further hybridization is impossible due to sterility). If the hybrids are fertile, then a mixture of hybrids of different generations and the results of backcrosses should be observed.

The reconstruction of karyotypes of pure forms of *P.orphicus* and *P.lurae* sp. nov and their putative hybrids is shown in Fig. 11. As follows from this scheme, F1 hybrids should all have the same number of elements visible in the first metaphase of meiosis (n=41), despite the fact that they may include 1 to 2 complex multivalents. Such a karyotype was not observed in the putative hybrid zone. From this we conclude that, likely, there is no complete reproductive isolation between *P.orphicus* and *P.lurae*, and the observed karyotypes n=43 are the result of repeated hybridization and backcrosses. Another hypothesis to explain the pattern we observe would be that *P.orphicus* lineage orph2 in Albania displays a karyotype n=42–43 and the specimens with n=43 are not admixed, although the fact that they were only found in the contact zone and that the two specimens measured displayed intermediate morphology favour the hybridisation hypothesis.

### ﻿Taxonomic position and difference from sister taxa

The data obtained demonstrate that *P.orphicus* and *P.lurae* represent two distinct phylogenetic lineages with a parapatric distribution. Indeed, both *P.orphicus* and *P.lurae* formed a highly supported monophyletic lineage based on three phylogenetic analyses (BI of *COI* barcode, ML of *COI*+*ITS2* and BI of *COI*+*ITS2*) (Fig. 2, Suppl. material 2). These two lineages are also substantially differentiated with respect to morphology (different wing reflectance), and karyotype (difference in one chromosome pair). Therefore, they can be considered species from the viewpoint of the phylogenetic species concept. These two lineages (=phylogenetic species) overlap in a small contact zone in Albania, and the combination of mtDNA, karyotype and morphological data suggest that they may hybridize and no complete barrier to reproduction exist.

Theoretically, the main lineages in the *P.orphicus*, *P.timfristos*, *P.aroaniensis* and *P.lurae* subcomplex could also be interpreted as infraspecific taxa, if the polytypic species concept is applied (Vishnevskaya et al. 2016). In this case, these taxa would be subspecies under the entire *P.aroaniensis* species complex. In Albania we showed that there is a contact zone between *P.orphicus* and *P.lurae* where unusual combinations of mitochondrial and karyotype, as well as intermediate morphotypes, exist. However, none of the aforementioned taxa appear to be fully sympatric in distribution and, taken together, they form a highly supported monophyletic lineage based on analysis of *COI* sequences (Fig. 2) and the concatenated *COI*+*ITS2* sequences (Suppl. material 2). Under this scenario, this subspecies-complex would be considered a diverse array of allopatric populations, each of which possesses unique genetic attributes (karyotypes and molecular markers) and is distributed in a particular area within the Balkan peninsular. While one can argue that differences in chromosome numbers in the subgenus Agrodiaetus do not necessarily result in complete reproductive isolation and, at least in some particular cases, do not prevent interspecific hybridization and genetic introgression (Lukhtanov et al. 2015b), this does not necessarily mean that chromosomal rearrangements are irrelevant to the formation of genetic barriers between populations (Vishnevskaya et al. 2016).

Chromosome changes have been shown to be important for speciation in Polyommatina butterflies (Lukhtanov et al. 2005; Kandul et al. 2007; Talavera et al. 2013a; Vishnevskaya et al. 2016) and even a weak reduction in fertility in heterozygotes for multiple chromosomal rearrangements can result in selection against them and in the formation of a boundary between chromosomally diverged homozygous populations. More detailed studies investigating lab-controlled crosses between sister taxa and the fertility of their progeny would be interesting to shed light on this topic, as has been achieved in wood white (*Leptidea*) butterflies (Dincă et al. 2013). Recent taxonomical publications have treated *P.orphicus*, *P.aroaniensis* and *P.timfristos* as species-level taxa (Eckweiler and Bozano 2016; Wiemers et al. 2018) and our study is following this rationale also for *P.lurae* sp. nov.

Regardless of its taxonomic status as a species or subspecies, *P.lurae* represents a unique entity within the genus *Polyommatus* that deserves additional study. A better understanding of its evolutionary history and its relationship with its unique biotope and related taxa may be helpful in understanding mechanisms of chromosomal diversification within the subgenus Agrodiaetus, and may further elucidate the biogeography of the south Balkan and Aegean regions.

### ﻿Conservation of the species and habitat

The Lurë region has become a National Park (Parku Kombëtar Lurë-Mali i Dejës) since 1966 to protect its ecosystems and biodiversity. Since 2018 by encompassing the entire section of Kunora e Lurës, its name has changed to Parku Kombëtar Lurë-Mali i Dejës, spanning an expanded area of 202.42 km^2^. Despite its conservation status the area suffered massive deforestation from illegal logging and forest fires that severely affected ecosystems and it is estimated that as much as 50% of the original Lura National Park has been destroyed (Rama 2018). Moreover, it is not fully covering important biotopes such as some of the *P.lurae* biotopes.

Next to this, the first author noticed that sheep overgrazing is also affecting the ecosystems. As *Onobrychis* plants are very palatable to sheep, heavy grazing limits the growth and expansion of *Onobrychis*, sometimes leading to the extinction of the plant (Lafranchis et al. 2007). While traditional grazing by sheep is beneficial, and can help in keeping open clearings, uncontrolled and overgrazing can have a devastating impact on butterflies, and other insects such as bees, which has been increasingly reported in different parts of Europe (Kruess and Tscharntke 2002; Potts et al. 2009; Verbrugge et al. 2022) and such an evolution in Albania would be dramatic for its biodiversity, especially for ecosystems harbouring unique species diversity.

The future of various endemic species of *Polyommatus* in Europe is strongly dependent on keeping open dry clearings at montane-subalpine levels where its foodplant is growing; This is the case for *P.orphicus* and *P.aroaniensis* in Greece but even so for *P.lurae* in Albania.

As a distinct taxonomic entity occupying a very restricted area linked to a unique biotope in Albania the newly described species should be considered a candidate on the list of protected species in Albania and the whole of Europe by adding to the European red list of Butterflies (Van Swaay et al. 2010; Maes et al. 2019).

In summary, the Lurë region harbours unique endemic flora and fauna, in addition to being home for the species here described, which is currently only found very restricted and locally. Therefore, the preservation of this habitat needs being ensured. This encompasses also control of human activities as illegal logging, burning and uncontrolled grazing by livestock, all major factors that have been identified contributing to butterfly decline in Europe (van Swaay and Warren 2006). As Albania is setting up programs to be member of be the European Union, installing adequate protection legislation for its biodiversity heritage (e.g. the EU Habitats Directive 92/43/EEC) will be needed.

## Supplementary Material

XML Treatment for Polyommatus lurae

## Supplementary materials

Supplementary material 1 Colour measurements of wing reflectance Data type morphological Explanation note Deatails on colour measurements (methodology, processing) and generated data are given. https://binary.pensoft.net/file/769928This dataset is made available under the Open Database License (http://opendatacommons.org/licenses/odbl/1.0/). The Open Database License (ODbL) is a license agreement intended to allow users to freely share, modify, and use this Dataset while maintaining this same freedom for others, provided that the original source and author(s) are credited.Laurian Parmentier, Roger Vila, Vladimir Lukhtanov

Supplementary material 2 Phylogeny of concatenated *COI*+*ITS2* sequences based on NJ and BI reconstructions Data type docx file https://binary.pensoft.net/file/769929This dataset is made available under the Open Database License (http://opendatacommons.org/licenses/odbl/1.0/). The Open Database License (ODbL) is a license agreement intended to allow users to freely share, modify, and use this Dataset while maintaining this same freedom for others, provided that the original source and author(s) are credited.Laurian Parmentier, Roger Vila, Vladimir Lukhtanov
