# Supplementary material for: ﻿Integrative analysis reveals cryptic speciation linked to habitat differentiation within Albanian populations of the anomalous blues (Lepidoptera, Lycaenidae, Polyommatus Latreille, 1804)
Source: Comp Cytogenet. 2022 Nov 15;16(4):211–42. doi: 10.3897/CompCytogen.v16.i4.90558 (PMC9836409; doi:10.3897/CompCytogen.v16.i4.90558)
Supplement: Supplementary material 1 — Colour measurements of wing reflectance [file comparative_cytogenetics-16-4-211_article-90558__-s001.pdf]

Supplementary information 1. Colour measurements of wing reflectance

Wing colour is an important trait for identification of butterflies and a species-specific characteristic (Bálint et al. 2012), an indicator of genetic variation (Wasik et al. 2014), and evidence of a changing population (Kertész et al. 2021). Observing fixed differences in wing colour of butterflies of different population can serve as a reliable tool for taxonomists for taxonomical identification (Bálint et al. 2010). Here we used colour measurements of dorsal wings of male *Agrodiaetus* to generate standardized RGB measurements of set specimens.

Colour measurement set up and processing of images

In our set-up a constant light source in a darkened room was used (3 Marbul® suspension light sources of 12 W, 955 lm, 3500 K) in a triangle position at 1 m above the specimen to obtain a reproducible and uniform light source and minimize shades. RGB pictures were made on specimens positioned at an angle of 20° to the equatorial to measure maximum light reflectance generated by wing scale structures. The spectral position of the reflectance maximum of such photonic nanoarchitectures depends on the nanoscale geometric dimensions of the elements building up the nanostructure and was based on earlier experience and method described by Kertész et al. (2021). Pictures were taken with a Canon 70D and 100mm macrolens with stabiliser.

To obtain a uniform colour zone, the intervein space – which showed most reflectance variability- of the inner postdiscal zone (only between M1 and CU2 cells) was used. Per measurement a circular wing zone of was blurred in Lightroom® and the average colour obtained was mapped on a disc. **Figures S1-S3** are visualising the different samples photographed (upper and hind wings) of *P. orphicus*, *P. lurae* **sp. nova** and two *P. lurare x orphicus* hybrid, respectively, with colour discs of the 3 measurements. *COI* results (*orph2* and *aroa3*) and karyotype (*orphicus*, *lurae*) were added to obtain the categories 'orphicus', 'lurae', and 'hybrid'. In the analysis, substrate type was also integrated as a factor, with three categories 'ophiolite', 'karst', and 'mixed'

On each uniform colour disc obtained (3 samples per wing), colour measurements were done generating exact RGB and HUE values using the colour picker tool. Averaged values per specimen were used for statistics (**Table S1**). Wing colour measurements were taken only from fresh samples (worn specimens and those with minimal damage on fringe were discarded) belonging to the *Polyommatus (aroaniensis)* species-complex collected from different Albanian localities (Valikardhë and Lurë regions), with habitats harbouring ophiolitic, karst and mixed substrates.

Posterior statistics was done running a permutational multivariate analysis of variance, using distance matrices with the Adonis call (Vegan package) in R (Oksanen et al. 2016) , given in the main text.

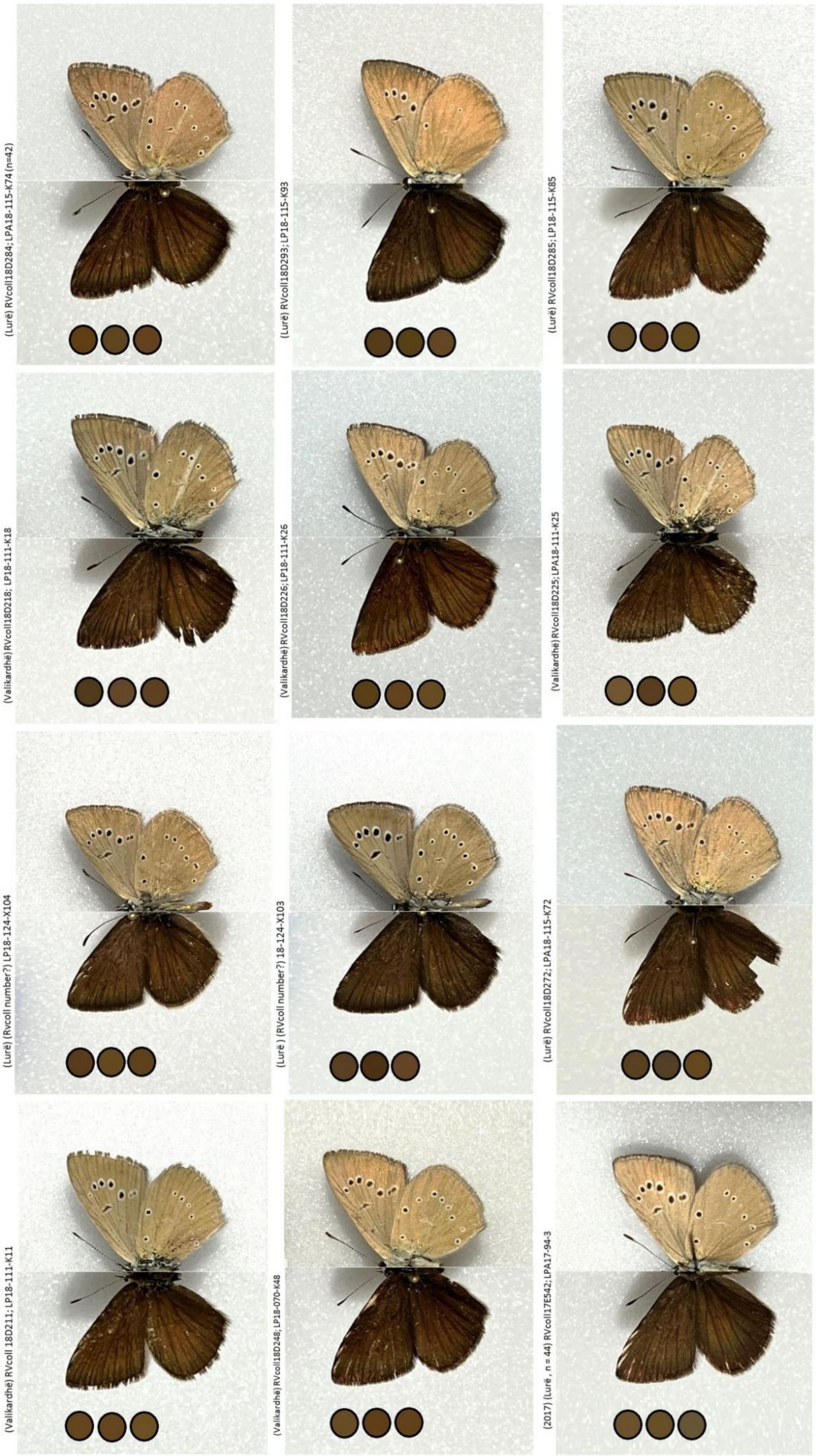

Figure S1. Composition of *P. orphicus* male samples of upper- and underwings photographs. Colour discs represent 3 independent uniform measurements of wing reflectance in the ups M1 and CU2 cells

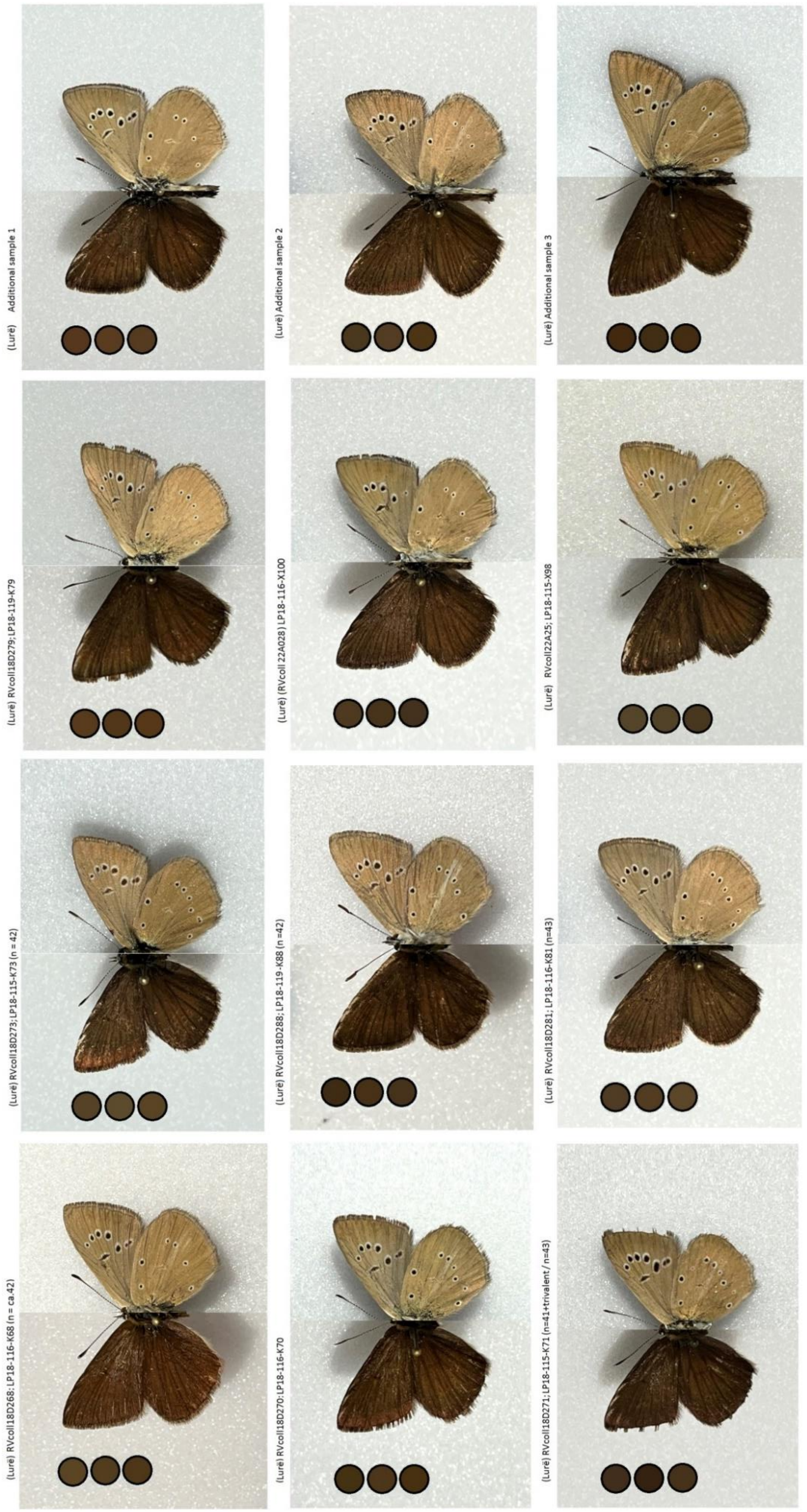

Figure S2. Composition of *P. lurae* **sp. nova** male samples of upper- and underwings photographs. Colour discs represent 3 independent uniform measurements of wing reflectance in the ups M1 and CU2 cells

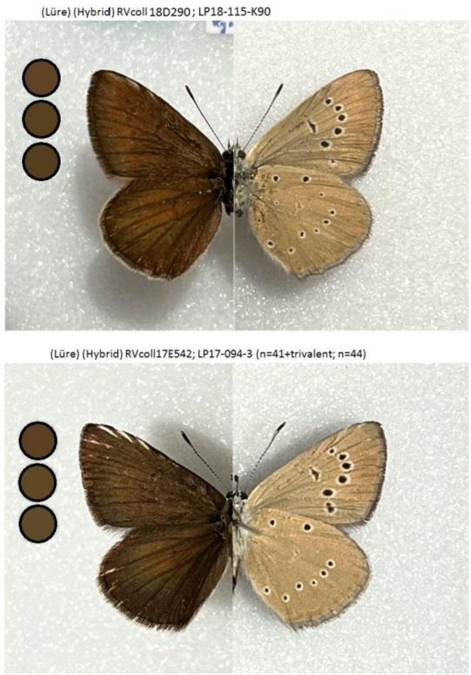

Figure S3. Composition of two putative hybrid *P. lurae x orphicus* male samples of upper- and underwings photographs. Colour discs represent 3 independent uniform measurements of wing reflectance in the ups M1 and CU2 cells

Table S1. Colour reflectance measurements (RGB and HSV values are given) on uniformised colour discs and averaged values used in the statistical analysis.

Sample n°	measure-ment	Values R	G	B	H	S	V	Average values R	G	B	H	S	V
orpicus (1)	1	102	70	31	32,9	70	40						
	2	102	70	31	32,9	70	40						
	3	108	81	36	37,5	67	42	104	74	33	34	69	41
orphicus (2)	1	105	77	39	34,5	63	41						
	2	96	62	24	31,7	75	38						
	3	98	66	27	32,9	72	38	100	68	30	33	70	39
orphicus (3)	1	108	82	45	35,2	58	42						
	2	103	79	40	37,1	61	40						
	3	102	86	54	40	47	40	104	82	46	37	55	41
orphicus (4)	1	81	59	29	34,6	64	32						
	2	101	72	41	31	59	40						
	3	94	65	33	31,5	65	37	92	65	34	32	63	36
orphicus (5)	1	89	64	25	36,6	72	35						
	2	99	66	30	31,3	70	39						
	3	103	75	35	35,3	66	40	97	68	30	34	69	38
orphicus (6)	1	112	87	49	36,2	56	44						
	2	91	62	32	30,5	65	36						
	3	111	81	38	35,3	66	44	105	77	40	34	62	41
orphicus (7)	1	89	60	27	31,9	70	35						
	2	102	74	31	36,3	70	40						
	3	94	66	27	34,9	71	37	95	67	28	34	70	37
orphicus (8)	1	91	65	35	32,1	62	36						
	2	77	50	20	31,6	74	30						
	3	100	70	39	30,5	61	39	89	62	31	31	66	35
orphicus (9)	1	89	65	33	34,3	63	35						
	2	84	64	39	33,3	54	33						
	3	101	72	29	35,8	71	40	91	67	34	34	63	36
orphicus (10)	1	95	63	19	34,7	80	37						
	2	94	70	33	36,4	65	37						
	3	101	65	29	30	71	40	97	66	27	34	72	38
orphicus (11)	1	86	60	25	34,4	71	34						
	2	87	66	23	40,3	74	34						
	3	100	72	35	34,2	65	39	91	66	28	36	70	36
orphicus (12)	1	96	73	35	37,4	64	38						
	2	96	66	32	31,9	67	38						
	3	99	75	31	38,8	69	39	97	71	33	36	67	38

Table S1 (continued). Colour reflectance measurements (RGB and HSV values are given) on uniformised colour discs and averaged values used in the statistical analysis.

Sample n°	measure-ment	Values						Average values					
		R	G	B	H	S	V	R	G	B	H	S	V
Lurae (1)	1	91	69	37	35,6	59	36						
	2	83	61	31	34,6	63	33						
	3	79	56	26	34	67	31	84	62	31	35	63	33
Lurae (2)	1	69	50	19	37,2	72	27						
	2	77	54	26	32,9	66	30						
	3	72	49	17	34,9	76	28	73	51	21	35	71	28
Lurae (3)	1	65	46	29	28,3	55	25						
	2	53	34	17	28,3	68	21						
	3	71	51	27	32,7	62	28	63	44	24	30	62	25
Lurae (4)	1	66	41	16	30	76	26						
	2	66	46	21	33,3	68	26						
	3	69	45	19	31,2	72	27	67	44	19	32	72	26
Lurae (5)	1	89	60	30	30,5	66	35						
	2	83	55	26	30,5	69	33						
	3	87	57	26	30,5	70	34	86	57	27	31	68	34
Lurae (6)	1	86	67	39	35,7	55	34						
	2	92	75	45	38,3	51	36						
	3	89	68	38	35,3	57	35	89	70	41	36	54	35
Lurae (7)	1	64	43	21	30,7	67	25						
	2	71	49	23	32,5	68	28						
	3	71	51	27	32,7	62	28	69	48	24	32	66	27
Lurae (8)	1	79	58	33	32,6	58	31						
	2	72	52	27	33,3	63	28						
	3	68	49	30	30	56	27	73	53	30	32	59	29
Lurae (9)	1	80	60	36	32,7	55	31						
	2	82	61	34	33,7	59	32						
	3	92	71	42	34,8	54	36	85	64	37	34	56	33
Lurae (10)	1	87	56	31	26,8	64	34						
	2	90	60	32	29	64	35						
	3	85	59	32	30,6	62	33	87	58	32	29	63	34
Lurae (11)	1	68	47	20	33,8	71	27						
	2	65	44	17	33,8	74	25						
	3	65	42	10	34,9	85	25	66	44	16	34	77	26
Lurae (12)	1	76	56	31	33,3	59	30						
	2	83	62	37	32,6	55	33						
	3	78	53	22	33,2	72	31	79	57	30	33	62	31
Hybrid (1)	1	94	68	37	32,6	61	37						
	2	88	65	29	36,6	67	35						
	3	84	63	30	36,7	64	33	89	65	32	35	64	35
Hybrid (2)	1	92	66	34	33,1	63	36						
	2	95	72	36	36,6	62	37						
	3	95	74	41	36,7	57	37	94	71	37	35	61	37

References

Bálint, Z., K. Kertész, G. Piszter, Z. Vértesy, and L. P. Biró. 2012. The well-tuned blues: the role of structural colours as optical signals in the species recognition of a local butterfly fauna (Lepidoptera: Lycaenidae: Polyommatinae). Journal of The Royal Society Interface **9**:1745-1756.

Bálint, Z., J. Wojtusiak, G. Piszter, K. Kertész, and L. Biro. 2010. Spectroboard: An instrument for measuring spectral characteristics of butterfly wings - A new tool for taxonomists. Genus **21**:163-168.

Kertész, K., Z. Bálint, G. Piszter, Z. E. Horvath, and L. P. Biro. 2021. Multi-instrumental techniques for evaluating butterfly structural colors: A case study on *Polyommatus bellargus* (Rottemburg, 1775) (Lepidoptera: Lycaenidae: Polyommatinae). Arthropod Structure & Development **61**.

Oksanen, J., F. G. Blanchet, R. Kindt, P. Legendre, P. R. Minchin, R. B. O'Hara, G. L. Simpson, P. Solymos, M. Henry, H. Stevens, and H. Wagner. 2016. Community Ecology Package 'Vegan'. https://cran.r-project.org/web/packages/vegan/vegan.pdf.

Wasik, B. R., S. F. Liew, D. A. Lilien, A. J. Dinwiddie, H. Noh, H. Cao, and A. Monteiro. 2014. Artificial selection for structural color on butterfly wings and comparison with natural evolution. Proceedings of the National Academy of Sciences **111**:12109-12114.
