# Supplementary material for: ﻿Integrative analysis reveals cryptic speciation linked to habitat differentiation within Albanian populations of the anomalous blues (Lepidoptera, Lycaenidae, Polyommatus Latreille, 1804)
Source: Comp Cytogenet. 2022 Nov 15;16(4):211–42. doi: 10.3897/CompCytogen.v16.i4.90558 (PMC9836409; doi:10.3897/CompCytogen.v16.i4.90558)
Supplement: Supplementary material 2 — Phylogeny of concatenated COI+ITS2 sequences based on NJ and BI reconstructions [file comparative_cytogenetics-16-4-211_article-90558__-s002.docx]

**Supplementary information 2**

The Bayesian analysis and NJ analysis based on the concatenated matrix *COI+ITS2*. The NJ analysis was performed using with Tamura3-parameter+G (Kumar et al., 2018) as the optimal model. The Bayesian analysis was performed using the program MrBayes 3.2 (Ronquist et al. 2012) with default settings. See main text, Methods section, for detailed methodology description.


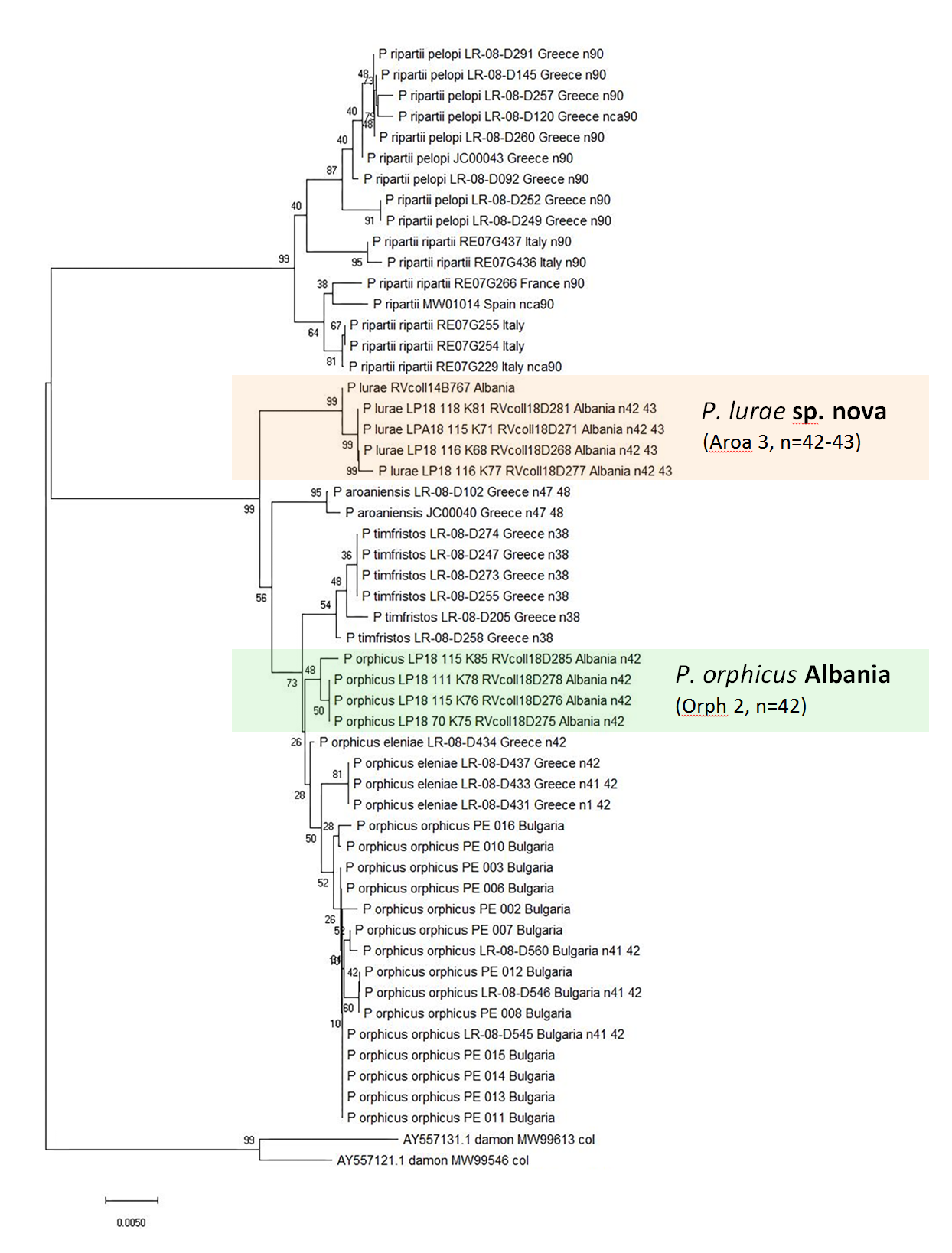


**S2, Figure 1**. NJ analysis of the concatenated matrix (using Tamura3+G as the best model). Coloured boxes are indicating *P. lurae* **sp. nova** (Aroa 2, n=42,43) and Albanian *P. orphicus* lineage (Orph 2, n=42).


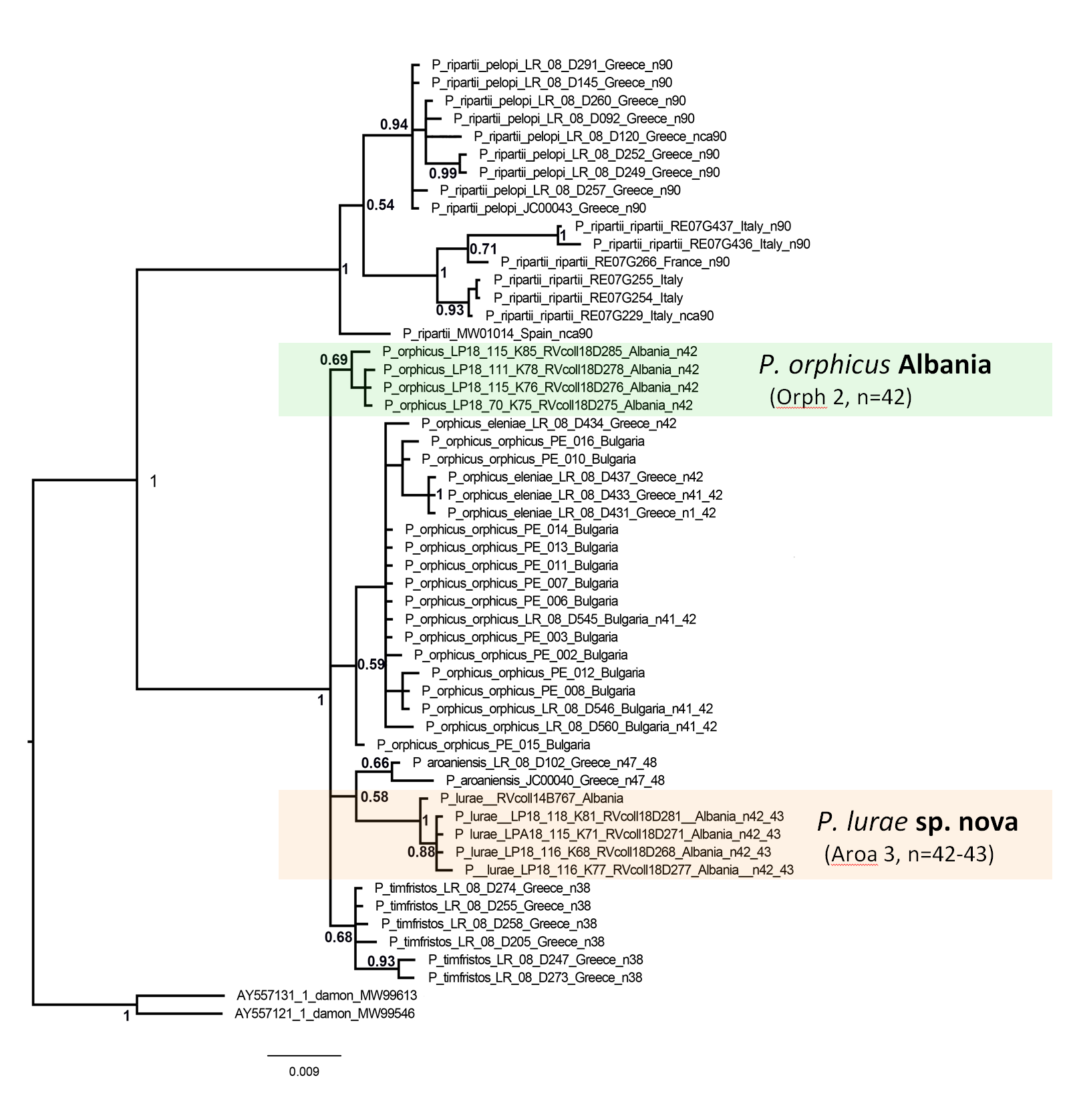


**S2, Figure 2**. BI analysis of the concatenated matrix generated in MrBayes 3.2 (using default settings). Coloured boxes are indicating *P. lurae* **sp. nova** (Aroa 2, n=42,43) and Albanian *P. orphicus* lineage (Orph 2, n=42).

**References**

Kumar S., Stecher G., Li M., Knyaz C., & Tamura K. 2018: MEGA X: Molecular Evolutionary Genetics Analysis across computing platforms. – Mol. Biol. Evol. 35: 1547-1549.

Ronquist F, Teslenko M, van der Mark P, Ayres DL, Darling A, Hohna S, Larget B, Liu L, Suchard MA, Huelsenbeck JP. 2012. MrBayes 3.2: efficient Bayesian phylogenetic inference and model choice across a large model space. Systematic Biology 61: 539–542. [doi: 10.1093/sysbio/sys029](https://doi.org/10.1093/sysbio/sys029)
